# Hybrid Equation/Agent-Based Model of Ischemia-Induced Hyperemia and Pressure Ulcer Formation Predicts Greater Propensity to Ulcerate in Subjects with Spinal Cord Injury

**DOI:** 10.1371/journal.pcbi.1003070

**Published:** 2013-05-16

**Authors:** Alexey Solovyev, Qi Mi, Yi-Ting Tzen, David Brienza, Yoram Vodovotz

**Affiliations:** 1 Department of Mathematics, University of Pittsburgh, Pittsburgh, Pennsylvania, United States of America; 2 Department of Sports Medicine and Nutrition, University of Pittsburgh, Pittsburgh, Pennsylvania, United States of America; 3 Center for Inflammation and Regenerative Modeling, McGowan Institute for Regenerative Medicine, University of Pittsburgh, Pittsburgh, Pennsylvania, United States of America; 4 Department of Rehabilitation Science and Technology, University of Pittsburgh, Pittsburgh, Pennsylvania, United States of America; 5 Department of Surgery, University of Pittsburgh, Pittsburgh, Pennsylvania, United States of America; University of Virginia, United States of America

## Abstract

Pressure ulcers are costly and life-threatening complications for people with spinal cord injury (SCI). People with SCI also exhibit differential blood flow properties in non-ulcerated skin. We hypothesized that a computer simulation of the pressure ulcer formation process, informed by data regarding skin blood flow and reactive hyperemia in response to pressure, could provide insights into the pathogenesis and effective treatment of post-SCI pressure ulcers. Agent-Based Models (ABM) are useful in settings such as pressure ulcers, in which spatial realism is important. Ordinary Differential Equation-based (ODE) models are useful when modeling physiological phenomena such as reactive hyperemia. Accordingly, we constructed a hybrid model that combines ODEs related to blood flow along with an ABM of skin injury, inflammation, and ulcer formation. The relationship between pressure and the course of ulcer formation, as well as several other important characteristic patterns of pressure ulcer formation, was demonstrated in this model. The ODE portion of this model was calibrated to data related to blood flow following experimental pressure responses in non-injured human subjects or to data from people with SCI. This model predicted a higher propensity to form ulcers in response to pressure in people with SCI vs. non-injured control subjects, and thus may serve as novel diagnostic platform for post-SCI ulcer formation.

## Author Summary

Pressure ulcers are costly and life-threatening complications for people with spinal cord injury (SCI). To gain insight into the pathogenesis and effective treatment of post-SCI pressure ulcers, we constructed a computer simulation in a hybrid modeling platform which combines both equation- and agent-based models. The model was calibrated using skin blood flow data and reactive hyperemia in response to pressure and predicted a higher propensity to form ulcers in response to pressure in people with SCI vs. non-injured control subjects. The methodology we present in the paper may eventually be used as a novel platform to study post-SCI ulcer formation, as well as serving as a framework for other biological contexts in which agent-based models and mathematical equations can be integrated.

## Introduction

In the United States, it is estimated that approximately 250,000 people live with spinal cord injury (SCI). Approximately 12,000 new cases occur each year [1], with total direct costs for treating all cases of SCI exceeding $7 billion annually [2], [3]. Pressure ulcers are common, costly and life-threatening complications for people with SCI. The prevalence of pressure ulcers in people with SCI is estimated to range from 8% to as high as 33% [4]. Post-SCI pressure ulcers are caused by a combination of impaired sensation, reduced mobility, muscle atrophy, as well as reduced vascularity and perfusion [5]. The current consensus is that pressure alone or pressure in combination with shear force cause localized injury to the skin and/or underlying tissue, usually over a bony prominence [6]. Several pathways have been identified for pressure/shear-induced ulceration, the major one being tissue ischemia.

Prolonged tissue ischemia may cause inflammation, necrosis, and the eventual formation of a pressure ulcer [7], [8]. Tissue inflammation is the common physiological reaction caused by tissue ischemia before necrosis occurs. We have focused our attention on this complex biological process. Inflammation is a central, modulating process in many complex diseases (e.g. sepsis, infectious disease, trauma, and wound healing), and is a central driver of the physiology of people with SCI [9]–[12]. However, inflammation is not an inherently detrimental process: properly regulated inflammation is required for successful immune response and wound healing [9], [13], [14]. Inflammation is a prototypical complex, nonlinear biological process that has defied reductionist, linear approaches [15]–[18]. Dynamic computational simulations, including ordinary differential equation (ODE)- and agent-based models (ABM), have been employed to gain insights into inflammation. These simulations have been useful in integrating mechanistic information and predicting qualitative and quantitative aspects of the inflammatory/wound healing response [19]–[22]. The purpose of the present study was to integrate blood flow data and the process of skin injury, inflammation, and healing using a hybrid model that combines ABM and ODE into a single computational model.

Agent-based modeling is an object-oriented, rule-based, discrete-event method of constructing computational models, and this technique can be used to model complex biological systems in which the behavior of individual components/agents, as well as pattern formation and spatial considerations are important [23]. Systems of ODE are well-suited for describing processes (or physiological responses) that can be approximated as well-mixed systems [22]–[26]. Modeling with differential equations (ordinary or partial) is the most widely used method of mathematical modeling. The main advantage of this approach is that there is a well-developed mathematical theory of differential equations which helps to analyze such equations and in some cases completely solve them [23], [25], [26]. To model a complex biological system as an ABM, the system is divided into small computational units (“agents”), with each agent obeying a set of rules that define the behavior of this agent. These simple rules, performed stochastically by agents in the model, lead to a complex, often emergent behavior of the system as a whole. In many cases, agents need only local information on the state of the system, rather than being affected by the global system state. As such, ABM's are particularly well suited to representing the transition between mechanisms at one scale of organization to behavior observed at another. The object-rule emphasis of an ABM greatly simplifies the process of model construction without loss of important features in the system, and also allows for modeling biological processes that are known to have both local and global features [23].

Our primary goal in this study was to gain translationally-useful insights into post-SCI pressure ulcer formation using dynamic, mechanistic computational modeling. However, several issues exist with the use of either ABM's or ODE's alone in modeling the pressure ulcer formation. It is difficult to analyze the output of ABM's in order to derive insights into qualitative regimes or primary drivers of outcome. In addition, simulating ABM's is more computationally intensive than simulating ODE-based models. On the other hand, real-life systems are often too complex to be modeled using only ODE, and the corresponding equation-based models may become too complicated to carry out practically useful results. Hybrid modeling is an emerging technique that involves combining diverse types of computational models into a single simulation [27]–[29]. In this approach, ODE can be used to define certain agent rules (low-level details), and ABM to describe the behavior of the high-level components of our system. In the present study, we utilized ODE to model properties tissue ischemia, and an ABM to model the stochastic, pressure-driven ulcer formation behavior in people with and without SCI. Using this approach, we find that a model calibrated with blood flow data predicted a higher propensity to form ulcers in response to pressure in SCI patients vs. non-injured control subjects.

## Methods

### Experiment of reactive hyperemia

The skin blood flow data used for computing the parameters of the differential equation model were collected from 12 adults (six with SCI and six without). This study was approved by the University of Pittsburgh Institutional Review Board (IRB# PRO08060015), and was carried out after obtaining informed consent from the participants. The age range of the subjects recruited for this study was 20–50 years old. The actual age in each group was: subjects with spinal cord injury (26, 27, 35, 35, 43, 48 years old); subjects without any neurological deficits (21, 25, 29, 35, 36, 44 years old). There was no statistically significant difference in age between the two cohorts of subjects (data not shown). For people with SCI, only those with ASIA [30], a scale for classification of spinal injury, grade A and B, one-year post-injury and non-ambulatory are recruited. The reactive hyperemic response was induced with 60 mmHg of pressure for 20 min on the sacral skin, with the participants lying on their stomach on a mat table. A laser Doppler probe was located at the center of the indenter to collect the skin blood flow. Instrumentation details are published previously [31]. A sample blood flow data collected in the experiment is demonstrated in Figure S1. The raw blood flow data of all tested subjects are provided in Dataset S1 and the plots of these data are shown in Dataset S2.

**Figure 1:**
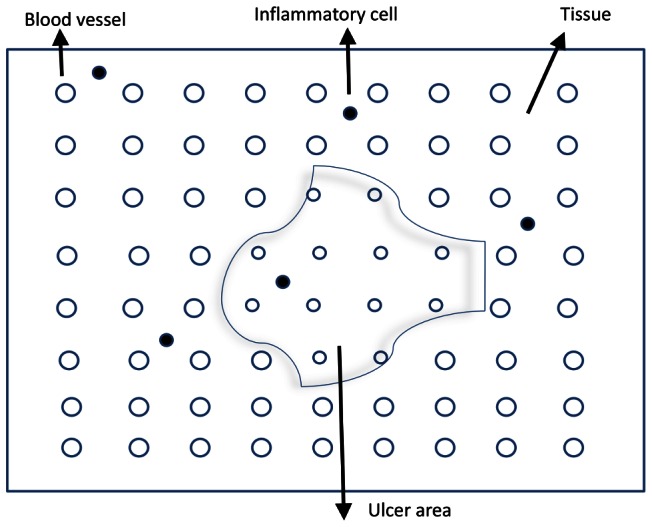
Hybrid model of pressure ulcer formation. The model representation of the pressure ulcer formation process is shown.

**Figure 2:**
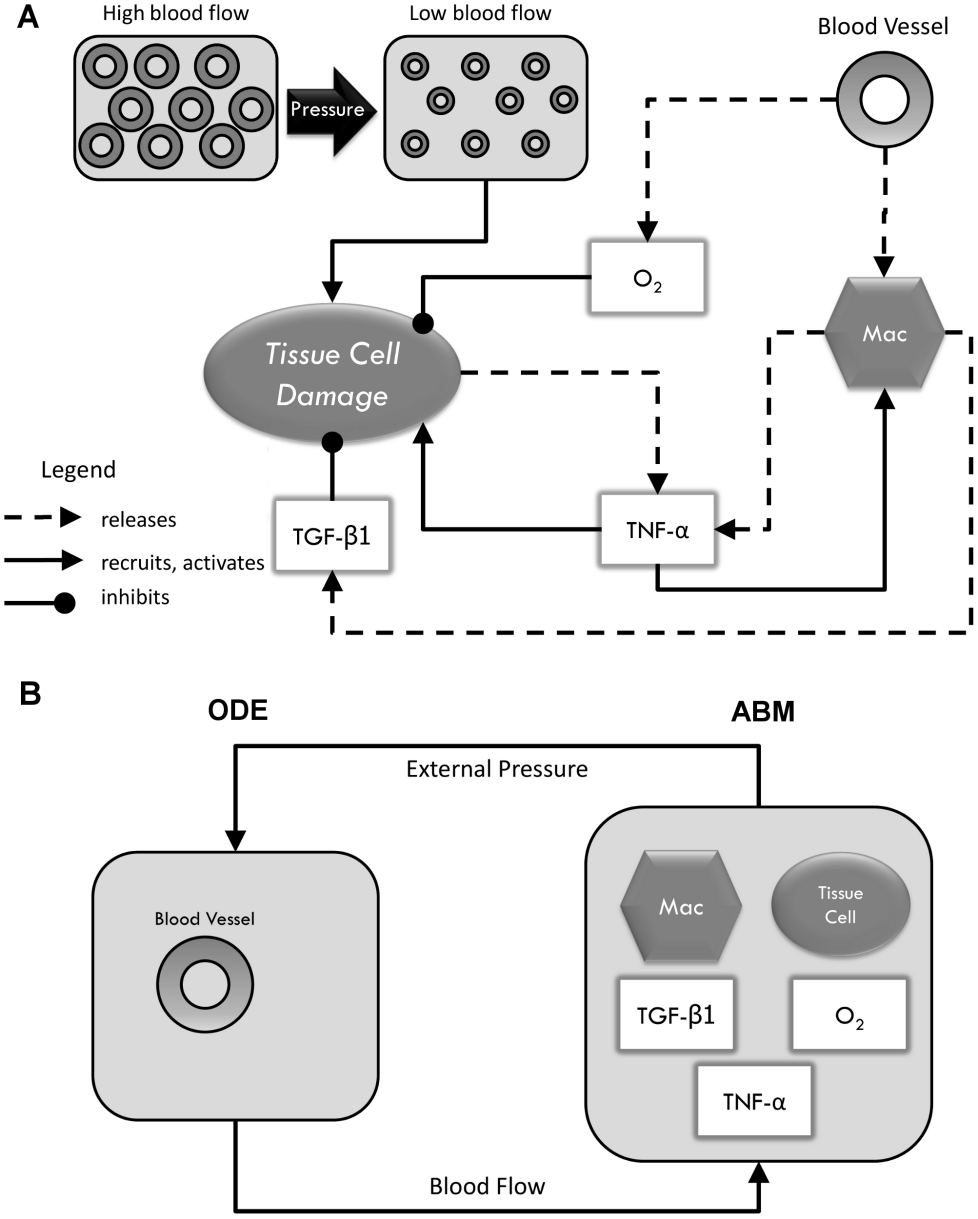
Components of the hybrid model of pressure ulcer formation. Panel (A) shows interactions between main components of the model. Panel (B) demonstrates the connection between ODE and ABM portions of the model. Geometric shapes represent model components. Arrows show interactions between components. “Mac” in the figure represents inflammatory cells (nominally macrophages).

The hybrid model utilized in our study is comprised of an ABM of skin/muscle injury, inflammation, and ulcer formation along with an ODE model of blood flow and reactive hyperemia. The ABM portion of the model comprises interactions among oxygen, pro-inflammatory elements, anti-inflammatory elements, and skin damage, with realistic predictions of the pattern, size, and progression of pressure ulcers. All rules of this ABM were generated based on literature reviews and previously-described ABM's of diabetic foot ulcer formation [21] and simplified pressure ulcer formation [32]. The ODE portion of the model simulates the ischemia-induced reactive hyperemic response, and is derived from a previous circuit model [33]. Figure 1 shows the model representation of the pressure ulcer formation. Figures 2A&B depict the model components and their interactions within the hybrid model, with the solid rectangles, ellipses and arrows representing the components of the ABM portion and the dashed ellipse and arrows representing the components of the ODE portion of the model.

### Agent-based model of pressure ulcer formation

The ABM portion of the model is based on our previously-developed models [21], [32]. This ABM is a simplified model that simulates inflammation and reactive hyperemic response (as the result of applied pressure) in a small segment of tissue (epithelial cells in the model). We implemented this ABM in SPARK (Simple Platform for Agent-based Representation of Knowledge; freely downloadable at http://www.pitt.edu/~cirm/spark) [34], following an extensive process of literature search and creation of graphical diagrams that incorporate known biological influences [20], [35], [36]. From such diagrams and based on our prior work on modeling of the formation of diabetic foot ulcers [21], we constructed rules by which individual agents (e.g. cells or cytokines) interact with each other and bring about biological effects. The ABM portion of the model consists of key cells and diffusible inflammatory signals assumed to be involved in the process of formation of a pressure ulcer. A similarly parsimonious approach was used to construct the rules and relationship among agents, with the goal of generating a high-level view of the process of pressure ulcer formation. The components and inter-relationships among the agents and variables of the pressure ulcer ABM are presented in Figure 2. Importantly, our model adheres to our prior work on the importance of the positive feedback loop of tissue damage/dysfunction→inflammation→tissue damage/dysfunction [22], [25].

The main components of the ABM portion of the model are: structural/functional skin cell (nominally epithelial cells); inflammatory cells (nominally macrophages); blood vessels; an aggregate pro-inflammatory cytokine agent (nominally TNF-α); an aggregate anti-inflammatory/pro-healing cytokine (nominally TGF-β1); and oxygen.

These agents interact according to the following rules. Epithelial cells are damaged by applied pressure. A damaged epithelial cell produces TNF-α. Epithelial cells also are damaged by excessive amount of TNF-α. A severely damaged epithelial cell dies. An epithelial cell can be healed by TGF-β1, and the healing rate is proportional to the amount of oxygen at the position of the epithelial cell.

Macrophages are attracted by TNF-α, and they also produce TNF-α and TGF-β1. Each macrophage has a fixed lifespan (measured in simulation steps) and a macrophage dies after several simulation steps.

Blood vessels create new macrophages and release oxygen. The rate of macrophage production and oxygen release depends on the amount of blood flowing through a blood vessel. The ODE portion of the model (see below) is incorporated into blood vessel rules, which specify how the oxygen is produced. Blood flow depends on the pressure applied on a blood vessel. A blood vessel dies if the surrounding epithelial cells die.

There are also global model rules which specify how oxygen, TNF-α, and TGF-β1 diffuse and evaporate.

Physical pressure in ABM portion of the model is applied periodically. More specifically, the pressure is applied for a fixed period of time. The pressure is then released for the same amount of time, and the process repeats. A specific model parameter (called Pressure Interval) specifies the pressure time interval.

A detailed description of ABM rules and parameters is given in Text S1.

### Ordinary differential equation model of ischemia-induced hyperemia

**Figure 3:**
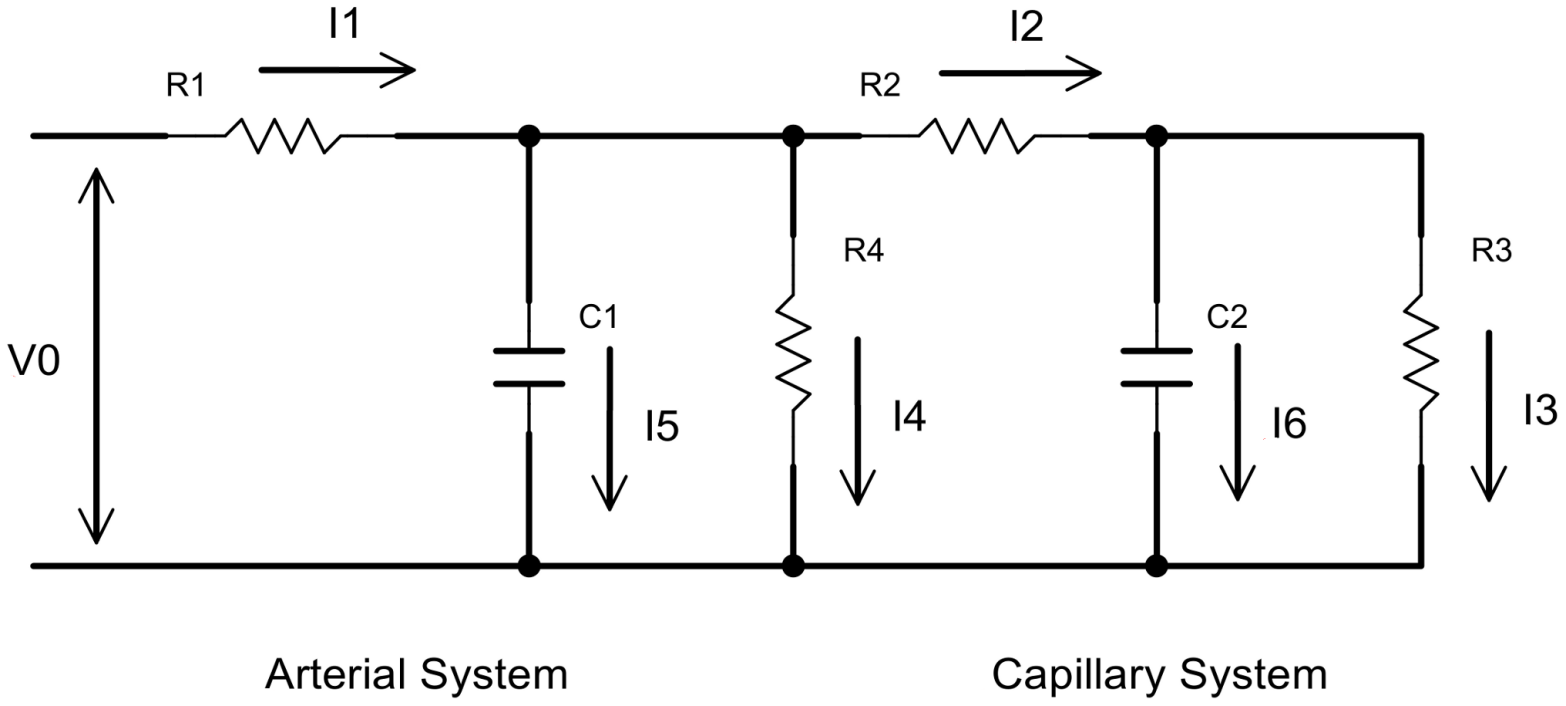
Circuit model of the blood flow. *R* (resistance) represents vascular resistance, *C* (capacitance) represents vessel compliance, *V*(*t*) represents the input blood flow pressure, and *I* (current) represents blood flow. *I*_2_(*t*) represents the skin blood flow.

Ischemia-induced hyperemia (the reactive hyperemic response) is a sudden increase in skin blood flow following tissue ischemia [37]. Hyperemia is a normal physiological response that can be easily induced with non-damaging ischemic events, and it has been used in numerous fields to examine endothelial function [38] and vascular activity [39]. We incorporated an ODE model of reactive hyperemia into the pre-existing ABM of ulceration in order to link measurable parameters of reactive hyperemia to the process of ulceration induced by repeated cycles of pressure and ischemia/reperfusion. To do so, we adopted the ODE-based circuit model of de Mul et al [40]. These authors suggested that the reactive hyperemic response could be modeled as the circuit shown at Figure 3, with *R* (resistance) representing vascular resistance, *C* (capacitance) representing vessel compliance, *V*(*t*) representing the input blood flow pressure, and *I* (current) representing blood flow. *I*_2_(*t*) represents the skin blood flow (specifically, reactive hyperemia) as measured using a laser Doppler flowmetry system.

The ODE system derived from the circuit model has the following form
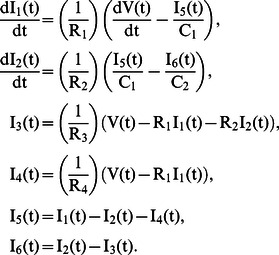
Note that here we have only two differential equations for *I*_1_(*t*) and *I*_2_(*t*). *I*_3_(*t*), *I*_4_(*t*), *I*_5_(*t*), and *I*_6_(*t*) can be algebraically eliminated.

We are interested in modeling a situation when an occlusion occurs in the input blood flow due to application of an external pressure. De Mul et al [40] model such a situation by considering the following stepwise input blood flow function
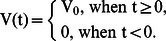
Here *V*_0_ is the aortic pressure. Based on this expression of *V*(*t*), an explicit solution for *I*_2_(*t*) can be derived with initial conditions *I*_1_(0) = *I*_2_(0) = 0. This solution has the following form
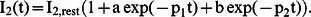
Here *I*_2,rest_, *a*, *b*, *p*_1_, and *p*_2_ are constants expressed in terms of *R*_1_, *R*_2_, *R*_3_, *R*_4_, *C*_1_, *C*_2_, *V*_0_.

We used this explicit solution for *I*_2_(t) for finding parameter values of the circuit model (the ODE portion of the model) based on available blood flow experimental data. In our agent-based simulations, the input blood pressure was a periodic function. In order to obtain the blood flow in these simulations, we used the ODE explicitly in our ABM.

### Model implementation in SPARK

The main components of SPARK models are *Space*, *Data Layers*, *Agents*, and the *Observer* [34]. *Space* is analogous to the physical space, and provides a context within which the model evolves. *Data Layers* provide a convenient way of tracking variables in space. *Data layers* update in time simultaneously at all positions. This is a computationally efficient way of handling processes such as diffusion and evaporation without employing an agent at each position to carry out the calculation. *Agents* can move, perform functions, interact with each other, and also interact with the space they occupy. Each agent has a set of behaviors and rules of action. The *Observer* contains information about space and all agents in the model. We extended SPARK with a feature for simple incorporation of ODE into an ABM. Epithelial cells, blood vessels, and macrophages were implemented as agents in SPARK. Oxygen, TNF-α, and TGF-β1 were implemented as data layers in SPARK. Pressure was implemented as a global model variable that periodically changes during the model simulation process.

The ODE portion of the model is integrated into the code of blood vessel agents. The following example shows how ODE's were added into SPARK-PL code:

equations

[

I4 = (V - R1 * I1)/R4

I3 = (V - R1 * I1 - R2 * I2)/R3

I5 = I1 - I2 - I4

I6 = I2 - I3

Dt I1 = (dV - I5/C1)/R1

Dt I2 = (I5/C1 - I6/C2)/R2

]

All variables in the example above are local variables of a blood vessel agent. Equations describe the evaluation of these variables in time. Each time step, the equation is integrated on the interval [*t*_1_, *t*_1_+*dt*], where *t*_1_ is the current simulation time and *dt* is the global parameter which specifies the time step. The output values of the equations are used in other rules defined for a blood vessel agent.

V represents the input blood pressure which is a periodic function in our simulations which depends on three parameters:
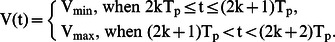
Here, *V*_max_ and *V*_min_ represent maximal and minimal blood pressures respectively; *T*_p_ is the pressure interval parameter of the model; *k* = 0,1,2, etc; *t* is the number of simulation ticks. In other words, we set *V* = *V*_min_ when the external pressure is applied and *V* = *V*_max_ when the external pressure is released. The SPARK source codes of this hybrid model are provided in Dataset S3.

## Results

### Fitting reactive hyperemia parameters

The ODE-based portion of the model was fit to data on blood flow for two different groups of subjects: a control group (CTRL) and an SCI group, as follows. We initially fixed parameters of the agent-based portion of the model. We chose these parameters based on a literature search. Only the approximate scale of parameters could be selected in this fashion, since our ABM is a simple, lumped-parameter model. With this set of parameter values, the ABM produces qualitative behavior commensurate with normal inflammation and wound healing [21].

**Figure 4:**
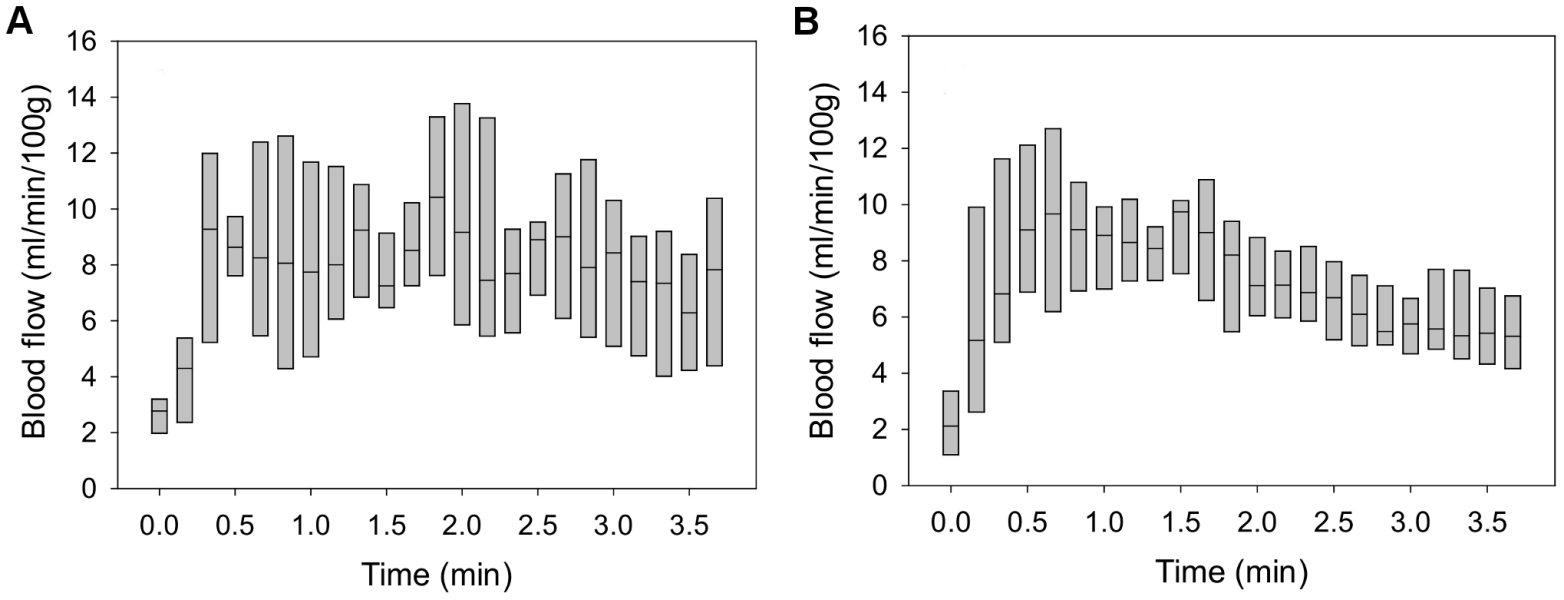
Average reactive hyperemia blood flow data. Raw blood flow data was filtered with low pass filters. The filtered data was averaged over all six subjects in each group. Panel (A) shows the averaged data for people with SCI. Panel (B) shows the averaged data for people without SCI.

Raw blood flow data was filtered with low pass filters. The filtered data were averaged over all six subjects in each group. Figures 4A and 4B depict the averaged reactive hyperemia blood flow data in people with and without SCI, respectively. We note that Figure 4A tend to oscillate more than Figure 4B. Depending on the level, and severity of injury, the reactive hyperemic response as measured with skin blood flow varied in people with SCI as compared to people without any neurological deficits. One main difference was the rate of increase and decrease in the skin blood flow of the reactive hyperemic response [41], in other words, one subject's peak blood flow may occur at 0.5 minute, and the other one may occur at 2.0 minute. With this variation, the blood flow oscillates more in Figures 4A as compared to Figures 4B. Another possible explanation is that, the skin blood flow as measured with the laser Doppler flowmetry system does oscillate naturally. When the skin blood flow signal was computed with Fourier transform, previous studies have identified that different frequency bands represent different physiological control mechanism of the blood flow [42]. Therefore the oscillation of skin blood flow is inevitable. We also note that the data in our simulation focused on the first 4 minutes. The interesting portion of the experimental data is the time when the peak blood flow occurs. We obtained approximately 10 minutes of raw data after releasing the pressure. The important information includes the time of the peak and the rate of decrease after the peak; both these values can be extracted from first 4 minutes after the pressure is released for all recorded data. We believe that it is simpler and more reliable to fit the ODE parameters based on the most important part of the experimental data (i.e. the first 4 minutes), since the rest of the data do not contain any important information for model fitting.

We then calibrated the ODE portion of the model based on the averaged data. Calibration was done using the following error function which measures the distance between actual (averaged) data and simulated results:
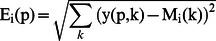
Here *i* is the group index, i.e., *i* is either CTRL or SCI. *E_i_* (*p*) is the error for the *i*-th group; *y*(*p*,*k*) is the value of the model function evaluated at the point *k* with the parameter vector *p*. *M_i_* (*k*) is the averaged *i*-th data at the point *k*.

Calibration was performed using Matlab R2011 (The Mathworks, Inc., Natick, MA, USA). We used the explicit expression of *I*_2_(*t*) for finding best-fit parameters. The values of *V*_max_ were assumed to be 85 mmHg for the control group and 75 mmHg for the SCI group, the same pressure values as in the experiments. For all other parameters, we defined possible lower and upper bounds. For the control group, we set 200 as the upper bound of all parameters, and 0.01 as the lower bound for all parameters except *R*_4_, for which we chose 190 as the lower bound since it is assumed that *R*_4_>>*R*_1_, *R*_2_, *R*_3_ [40]. Then we randomly selected 1000 points in the space of parameters and ran the standard Matlab minimization function fminsearch for all these initial points, and picked the best fit results. The search of best-fit parameters for the SCI group was carried out in a similar way. The only differences were that the value of *V*_max_ = 75, and in addition we changed the upper bounds of *C*_1_ and *C*_2_ and set them equal to the best-fit values of *C*_1_ and *C*_2_ for the control group. This change was made to reflect the fact that *C*_1,2_^SCI^<*C*_1,2_^CTRL^ [43].

**Figure 5:**
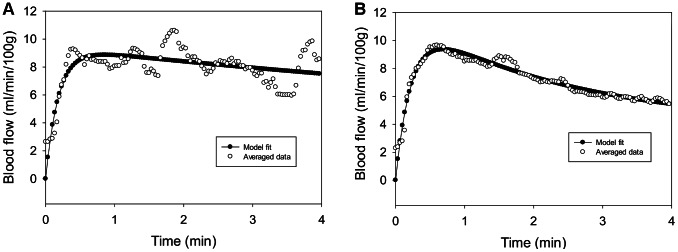
Fitted results for average reactive hyperemia. The explicit expression of *I*_2_(*t*) was used for finding best-fit results. Calibration was performed using Matlab R2011. Panel (A) shows the best-fit result in people with SCI. Panel (B) shows the best-fit result in people without SCI.

**Figure 6:**
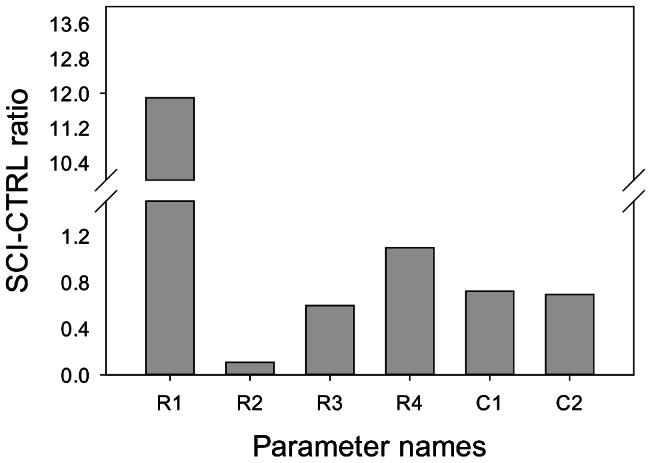
Best-fit parameter ratios for the differential equation part of the model. The x-axis shows the parameter names, and y-axis shows the ratios of parameters for people with and without SCI. The explicit expression of *I*_2_(*t*) was used for finding best-fit parameters.

Figures 5A and 5B show the best-fit simulation results, which minimize the error function *E_i_* (*p*) in data from people with and without SCI, respectively. Table 1 lists the values of the best-fit parameters for both group with the ratios calculated in the Figure 6 to show the significant change of parameters for people with and without SCI. The results show that vascular resistance (*R*_1_) is significantly increased and that blood vessel compliance (*C*_1_, *C*_2_) is decreased in the SCI group by comparing with the control group.

**Table 1:** Values of the best-fit parameters for control and SCI groups.

Group	*R*1	*R*2	*R*3	*R*4	*C*1	*C*2	*V*0
Control	0.10	7.49	13.83	191.10	69.28	14.45	85
SCI	1.19	0.80	8.28	210	50	10	75

The value of *V*_max_ was assumed to be 85 mmHg for the control group and 75 mmHg for the SCI group (these values are based on averaged experimental data). Then a standard Matlab minimization function was used to find the best fit results for all other parameters based on the explicit expression of *I*_2_(*t*).

### Hybrid model simulations suggest a greater propensity to ulcerate in SCI patients vs. controls

We next sought to determine the behavior of our simulation under a more clinically realistic setting, in which pressure to tissues alternates with periods of pressure relief. We also sought to determine if, once partially calibrated with blood flow data from control vs. SCI subjects, our model would predict differential propensity to ulcerate between these two groups of patients. We simulated the application of medium-scale pressure on the skin with different frequencies, first applying a pressure on the skin for a given period of time (pressure interval), releasing the pressure for the same amount of time, and then repeating the process. Using the parameters obtained as described above, we ran the model simulations for both groups and compared the outcome. We ran the model for 2000 steps with various values of the pressure interval parameter. All other ABM parameters were fixed. We assumed *V*_max_ = *V*_0_ (i.e., *V*_max_ = 85 for the control group and *V*_max_ = 75 for the SCI group) and *V*_min_ = 40 for both groups.

**Figure 7:**
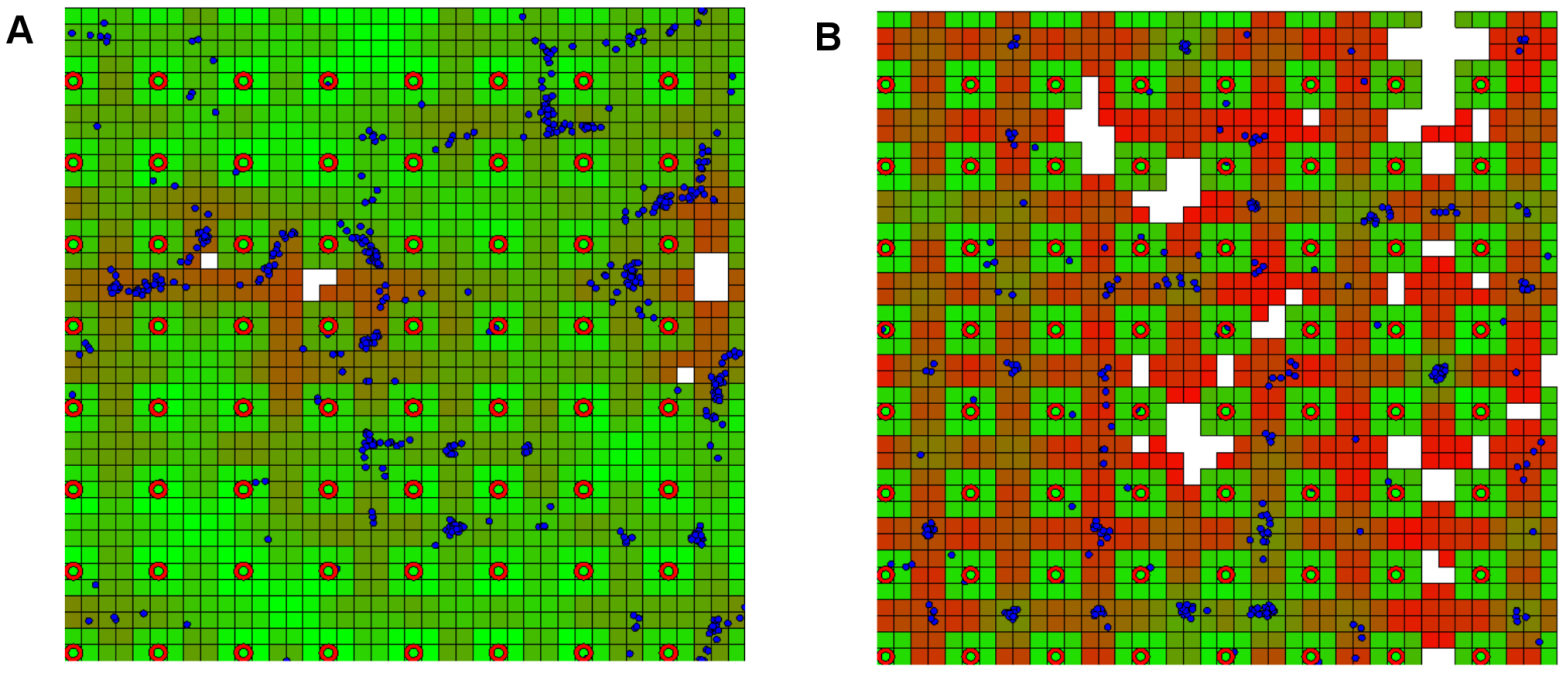
Simulation snapshots after 2000 steps. Green squares represent healthy epithelial cells, red squares represent damaged epithelial cells, red circles represent blood vessels, blue circles represent macrophage, and white squares represent the dead cells. Panel (A) shows the simulation result for the control group with the pressure interval = 210. Panel (B) shows the simulation result for the SCI group with the pressure interval = 107.

We initially examined the minimal value of the pressure interval that would be predicted to result in substantial tissue damage (death of some epithelial cell agents). Figures 7A and 7B show the SPARK simulation results for control and SCI subjects. Green squares represent healthy epithelial cells, red squares represent damaged epithelial cells, red circles represent blood vessels, and blue circles represent macrophage. For the control group, the minimal value of the pressure interval was 205–210 simulation ticks (Figure 7A); in contrast, for the SCI group, the minimal value was 105–110 simulation ticks (Figure 7B). We also performed subject-specific fitting of the ODE parameters and measured the minimal value of the pressure interval resulting in substantial tissue damage for each subject. The results are given in Table 2. The average subject-specific value of the minimal pressure interval was 207 for control subjects and 168 for SCI subjects. These results agree qualitatively with our findings for the averaged data presented above: the minimal pressure interval is larger for the control group.

**Table 2:** Subject-specific minimal value of the pressure interval that would be predicted to result in substantial tissue damage.

Group	*Subject ID*	*Pressure interval*
Control	C1	170
Control	C6	240
Control	C11	140
Control	C13	170
Control	C14	250
Control	C16	270
SCI	A7	240
SCI	A10	140
SCI	A14	140
SCI	A15	170
SCI	B3	180
SCI	B5	140

**Figure 8:**
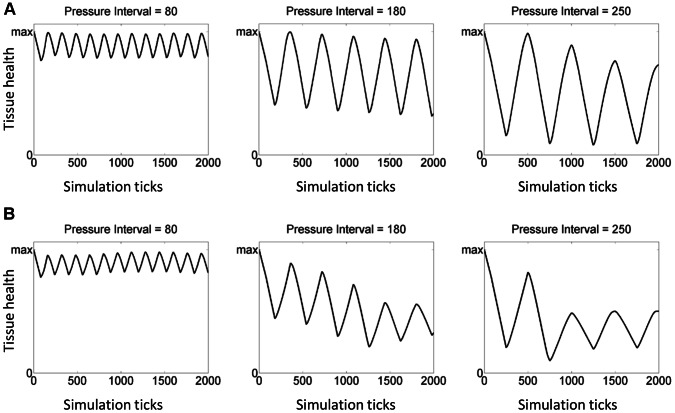
Simulation of different pressure scales on the health of epithelial cells. Graphs with different values of the pressure interval show how the predicted health of epithelial cells progresses over time for simulations of the control and SCI groups, respectively. Panel (A) shows the outcome of simulations for the control group. Panel (B) shows the outcome of simulations for the SCI group.

We next examined the predicted effect of turning frequency on control and SCI subjects. Figures 8A and 8B show how the predicted health of epithelial cells progresses over time for simulations of the control and SCI groups, respectively, over varying pressure on/off cycles. Increasing the frequency (or applying pressure for a short period of time and then subsequently relieving this pressure), we obtained an outcome in which a pressure ulcer did not form: when the simulated pressure is applied, the tissue is damaged somewhat, but when the pressure is relieved tissue health is restored. Also, simulated damage/dysfunction was predicted to increase more rapidly in the SCI group vs. the control group when the pressure interval was increased.

## Discussion

The components of the inflammatory response are time-driven, highly interconnected, and interact in a nonlinear fashion [15]–[18], [44]. The systems biology community has integrated mathematical and simulation technologies to understand complex biological processes [45]. More recently, we have suggested *translational systems biology* as a framework in which computational simulations are designed to facilitate *in silico* clinical trials, simulations are appropriate for *in vivo* and specifically clinical validation, and mechanistic simulations of whole-organism responses could guide rational therapeutic approaches [25].

Agent-based models have emerged as a useful complement to ODE-based models for elucidating complex biological systems, including inflammation, wound healing, angiogenesis, and cancer [19], [21], [23], [36], [46]–[49]. In the present study, we utilized a hybrid modeling approach that combines the both features of ODE and agent-based models. Using this approach, we integrate data regarding blood flow properties in SCI patients and compare them to data from control subjects. Our analysis suggests that, based on an abstraction of these blood flow properties and a stochastic model of tissue inflammation and ulcer formation, and in agreement with the literature [50], SCI patients are predicted to be more prone to ulceration. Our study, along with prior work [28], [51], [52], suggests that such hybrid modeling methodology could have a wide application in modeling complex, multiscale biological systems.

Despite the lack of sensation and motor function after SCI, several physiological changes at the chronic stage of SCI (more than 12 months since injury) increase a person's susceptibility to develop pressure ulcers, including changes in body composition (increased proportion of fatty tissue) and vascularity [5]. The linkage between changes in vascularity, epithelial function and pressure ulcer formation in people with SCI is not fully explored. Therefore, this pilot hybrid model was aimed at simulating pressure ulcer development by including a key vascular response (reactive hyperemia) observed in human subjects.

The goal of our previous research was to find the optimal turning frequency for patients with SCI [32]. The goal of the present model is the improvement of our previous model by coupling an ODE model of the reactive hyperemic response observed experimentally to an ABM based on rules derived from the literature. This model was capable of simulating the intensity in epithelial cell damage as a function of changes of amount and duration of localized pressure on the skin of people with and without SCI.

Results from the best-fit parameters of the circuit model set showed differences in vascular resistance (*R*_1_) and blood vessel compliance (*C*_1_, *C*_2_) between the two groups. The arterial resistance was bigger while the capillary resistance was smaller, respectively, in subjects with SCI as compared to controls. Changes in vascularity in people with SCI may be caused by denervation of sympathetic nervous system [53] as well as physical inactivity [54]. Our finding of increased vascular resistance in the arterial system was consistent with previous studies. With the loss of supraspinal control of the vascular system after high level of injury, people with SCI were reported to have increased vascular resistance in order to maintain the vascular tone by compensating for the loss of supraspinal sympathetic control [55]. Additionally, the increased vascular resistance may result from preservation of α-adrenergic tone. The increased vascular resistance could also result from vascular adaptation to deconditioning with the loss of motor function [56]. One prior study found that there was an increased activation of the receptor of the endothelin-1, which increases the vascular tone [56]. The results of decreased vascular resistance in the capillary system were not consistent with observations regarding vascular resistance in the arterial system. The capillary resistance was not investigated in previous studies; thus, our findings regarding vascular resistance in the arterial system may not be generalized to the capillary resistance, since the vascular resistance was measured with venous occlusion plethysmography in previous studies and the measurement was not directly on capillary blood flow. In addition, the measurement of reactive hyperemia in our study was at the lower back using an indenter, whereas the aforementioned previous studies measured this response at lower limbs with cuff. Future study on structural changes in capillary system and vascularity of the microcirculation might be beneficial in understanding the linkage to ulceration.

Results from the analysis of the best-fit parameters of the circuit model set also showed that the vessel compliance is smaller in people with SCI as compared to the controls. De Groot et al. found that the femoral artery compliance is smaller in individuals with SCI [43], and they suggested that this physiological change may be due to inactivity of the muscle since arterial compliance could be enhanced with functional electrical stimulation.

Our model validation studies suggest that the minimal amount of repeated pressure required to cause endothelial cell damage would be smaller in subjects with SCI. People with SCI are susceptible to ulcer formation, and there are several physiological changes that may contribute to the susceptibility of pressure ulcer development in this population. For example, people with complete SCI had decreased cross-sectional area of muscle fibers [57] and increased fat mass in lower limbs [58]. A recent study from Linder-Ganz et al. directly pointed out the relationship between physiological changes after injury and the pressure ulcer formation by using finite element model. They found that with the use of the same seat cushion, people with SCI had greater deep muscle stress as compared to controls [59]. To date, there is no study that investigated the direct linkage between changes in vascularity and ulcer formation in people with SCI. We were not aware of the underlying mechanism of changes in vascularity and the ulcer formation. However, from the rules and results of our model, it is indicated that changes in vascularity may play a role in decreased tolerance of pressure and endothelial function that leads to more severe damage with the same amount and duration of pressure.

There are several limitations of this study. This study only recruited limited numbers of subjects (six CTRL and six SCI), and people with SCI and controls were not matched for comparison. If additional subjects were used for the model calibration, the conclusion could be reached at a higher level degree of confidence. Though the ages of the subjects in the cohorts were not identical, there was no statistically significant difference with regard to age between the two groups of patients. In addition, previous studies [60], [61] found that the reactive hyperemic response was not different between healthy elderly population and healthy adults; these authors only found an impaired reactive hyperemic response among individuals in a hospitalized elderly population. Since there was no statistically significant difference in age between non-injured and SCI-injured subjects in our studies, and since all subjects recruited in our studies were healthy and not hospitalized during the time of the study, age is unlikely to be a significant factor in our data analysis. This is a pilot study developing this hybrid model of ulcer formation with different input of people with and without SCI. For a more realistic simulation, the ABM portion of the model could be expanded by incorporating additional physical and biological components, such as shear force and reperfusion injury, which may contribute to the formation of the pressure ulcer. Nevertheless, in this work, we present a first attempt to construct a biological model in a single computational platform where mathematical and agent-based models work in a seamless manner, and the result of the model reveals useful insight into the ulceration in people with and without SCI.

In conclusion, we used a hybrid approach combining ordinary differential equations related to blood flow along with an agent-based model of skin injury and subsequent inflammation in a single modeling platform, in order to investigate pathogenesis difference between people with SCI and without SCI in the process of ulcer formation. Our current finding suggests that people with SCI have higher propensity to form ulcers in response to pressure than non-injured control subjects.

## Supporting Information

Dataset S1 **Raw blood flow data of reactive hyperemia experiment for all subjects.**
(MAT)

Dataset S2 **Plots of the blood flow data of reactive hyperemia experiment for all subjects.**
(ZIP)

Dataset S3 **SPARK source codes of the hybrid model.**
(ZIP)

Figure S1 **A sample reactive hyperemia experiment blood flow profile.**
(TIF)

Text S1 **A detailed description of rules and parameters for the agent-based portion of the model.**
(DOCX)
