# Supplementary figures and images for: Hybrid Equation/Agent-Based Model of Ischemia-Induced Hyperemia and Pressure Ulcer Formation Predicts Greater Propensity to Ulcerate in Subjects with Spinal Cord Injury
Source: PLoS Comput Biol. 2013 May 16;9(5):e1003070. doi: 10.1371/journal.pcbi.1003070 (PMC3656105; doi:10.1371/journal.pcbi.1003070)

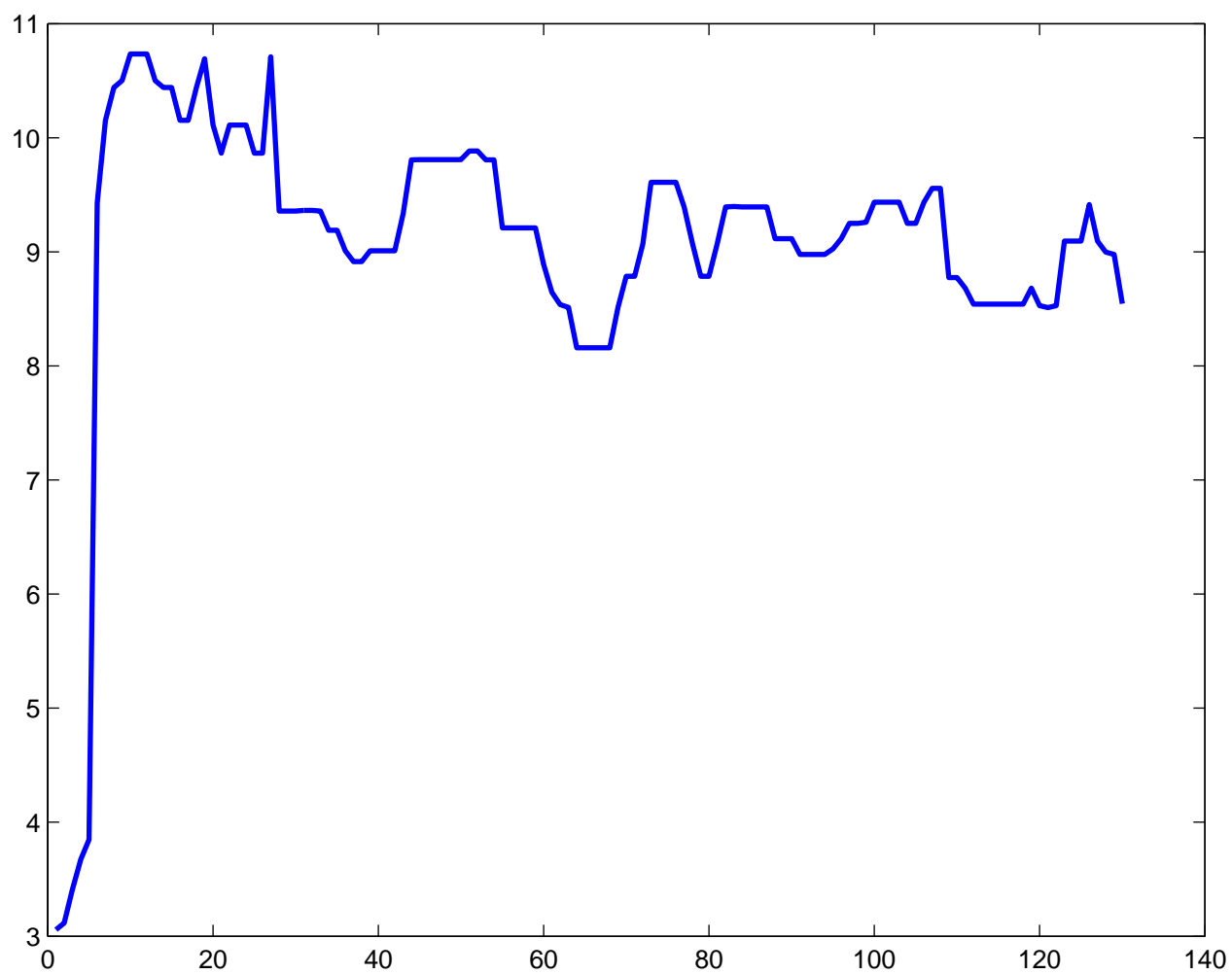

Supplement: Dataset S2 — Plots of the blood flow data of reactive hyperemia experiment for all subjects. (ZIP) [file pcbi.1003070.s002.zip › data/CTRL_C11_filt.pdf]

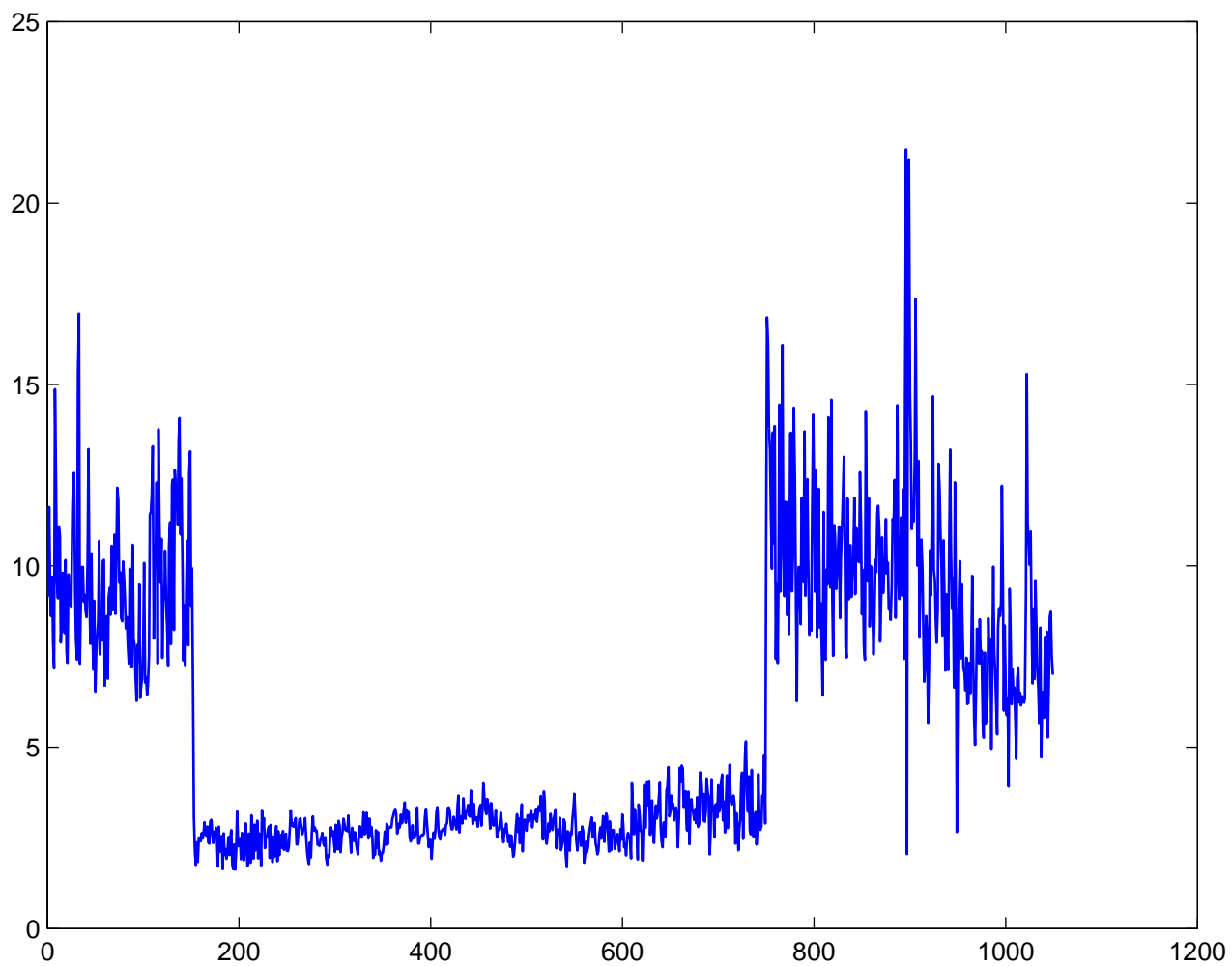

Supplement: Dataset S2 — Plots of the blood flow data of reactive hyperemia experiment for all subjects. (ZIP) [file pcbi.1003070.s002.zip › data/CTRL_C11_raw.pdf]

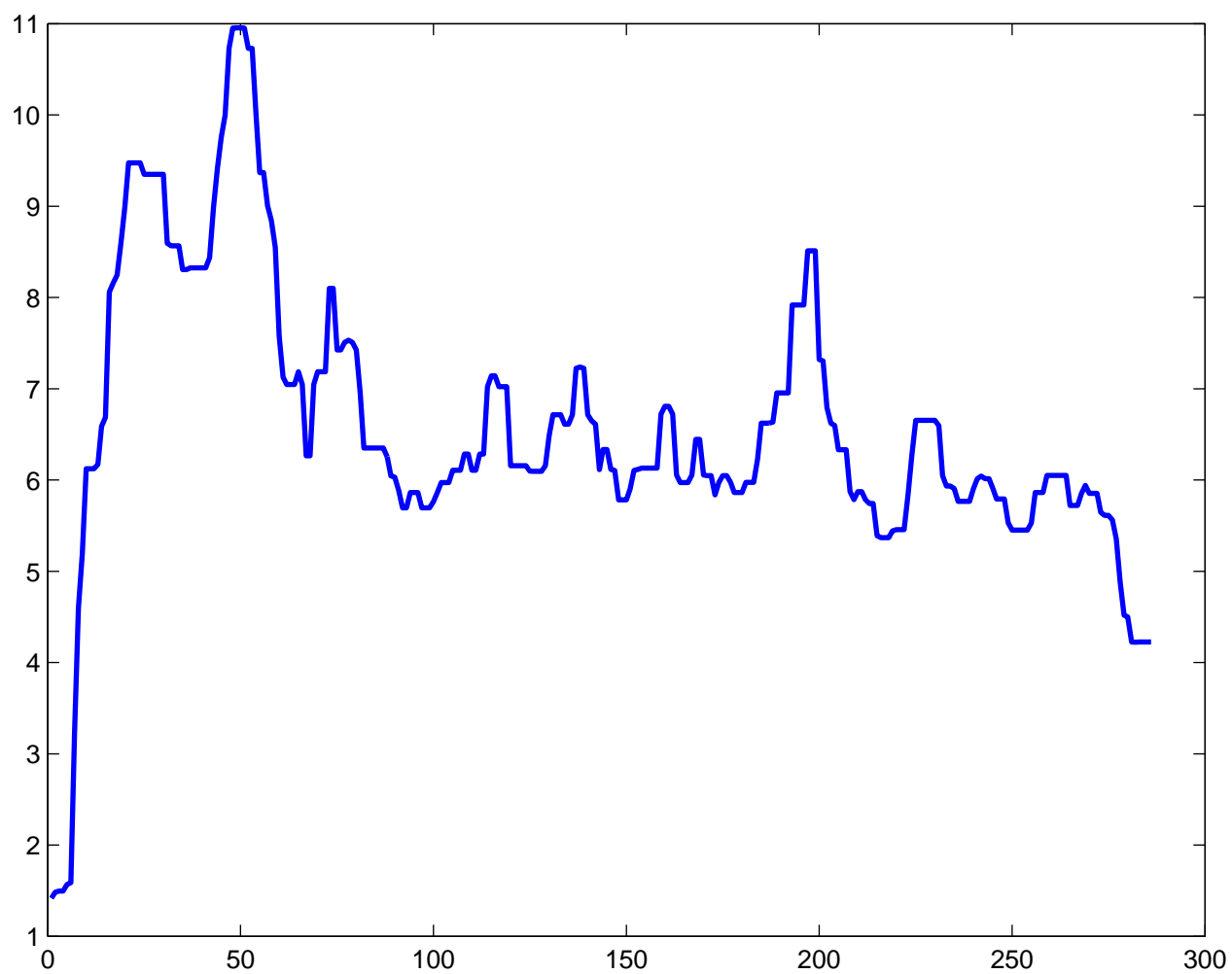

Supplement: Dataset S2 — Plots of the blood flow data of reactive hyperemia experiment for all subjects. (ZIP) [file pcbi.1003070.s002.zip › data/CTRL_C13_filt.pdf]

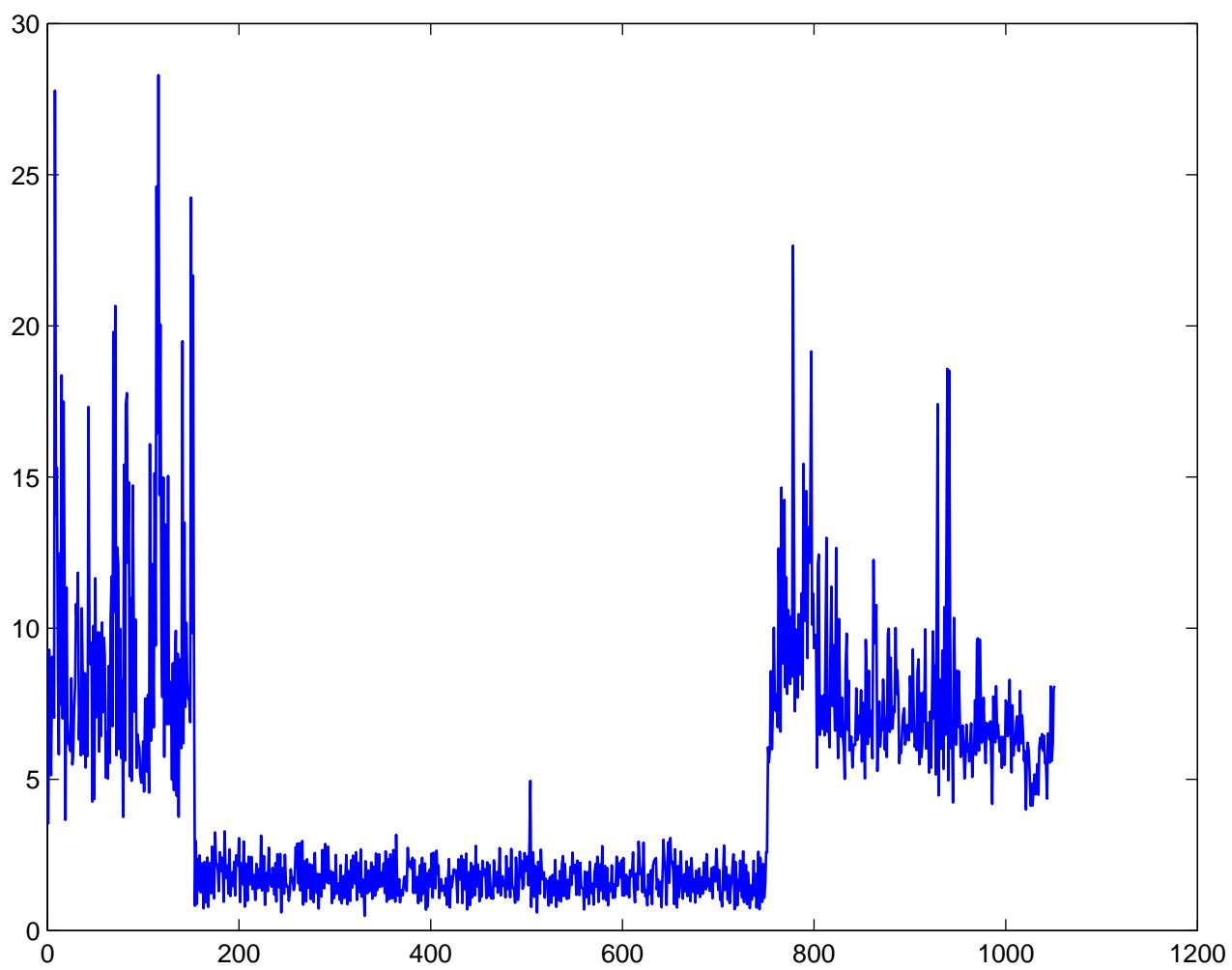

Supplement: Dataset S2 — Plots of the blood flow data of reactive hyperemia experiment for all subjects. (ZIP) [file pcbi.1003070.s002.zip › data/CTRL_C13_raw.pdf]

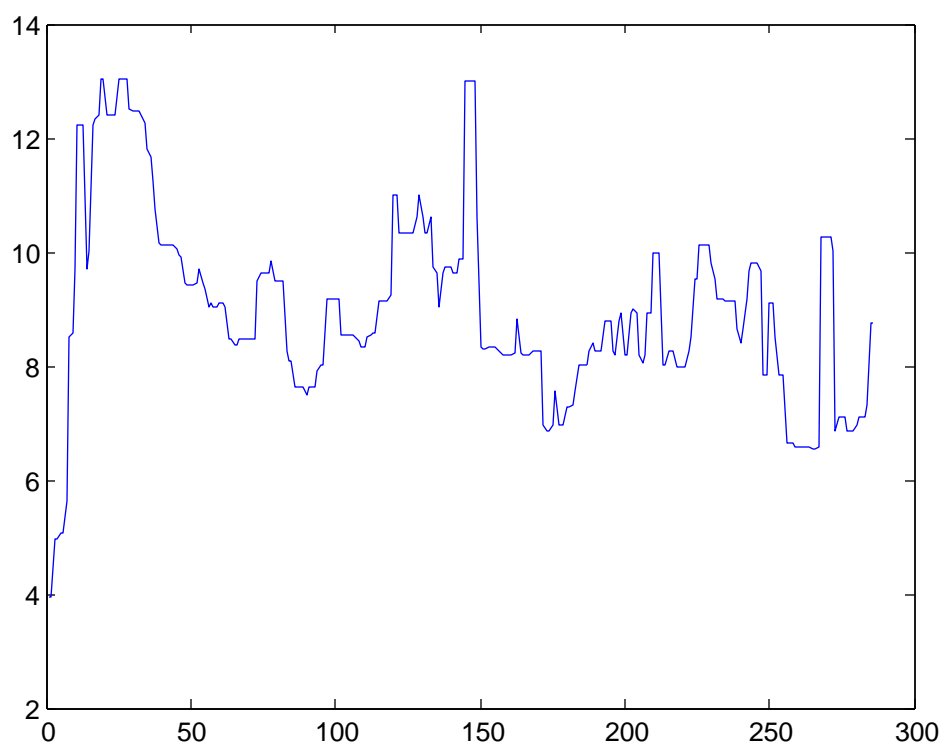

Supplement: Dataset S2 — Plots of the blood flow data of reactive hyperemia experiment for all subjects. (ZIP) [file pcbi.1003070.s002.zip › data/CTRL_C14_filt.pdf]

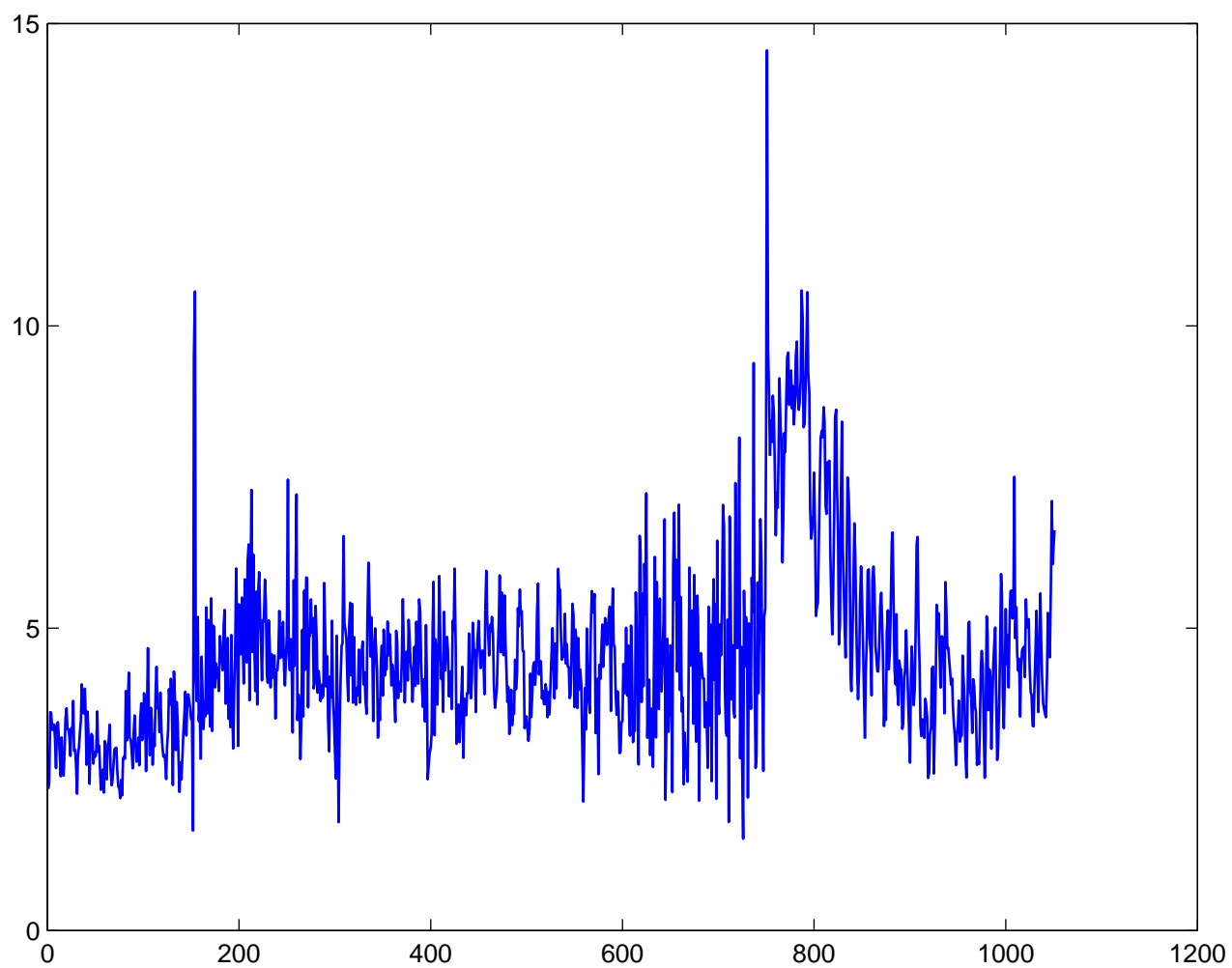

Supplement: Dataset S2 — Plots of the blood flow data of reactive hyperemia experiment for all subjects. (ZIP) [file pcbi.1003070.s002.zip › data/CTRL_C14_raw.pdf]

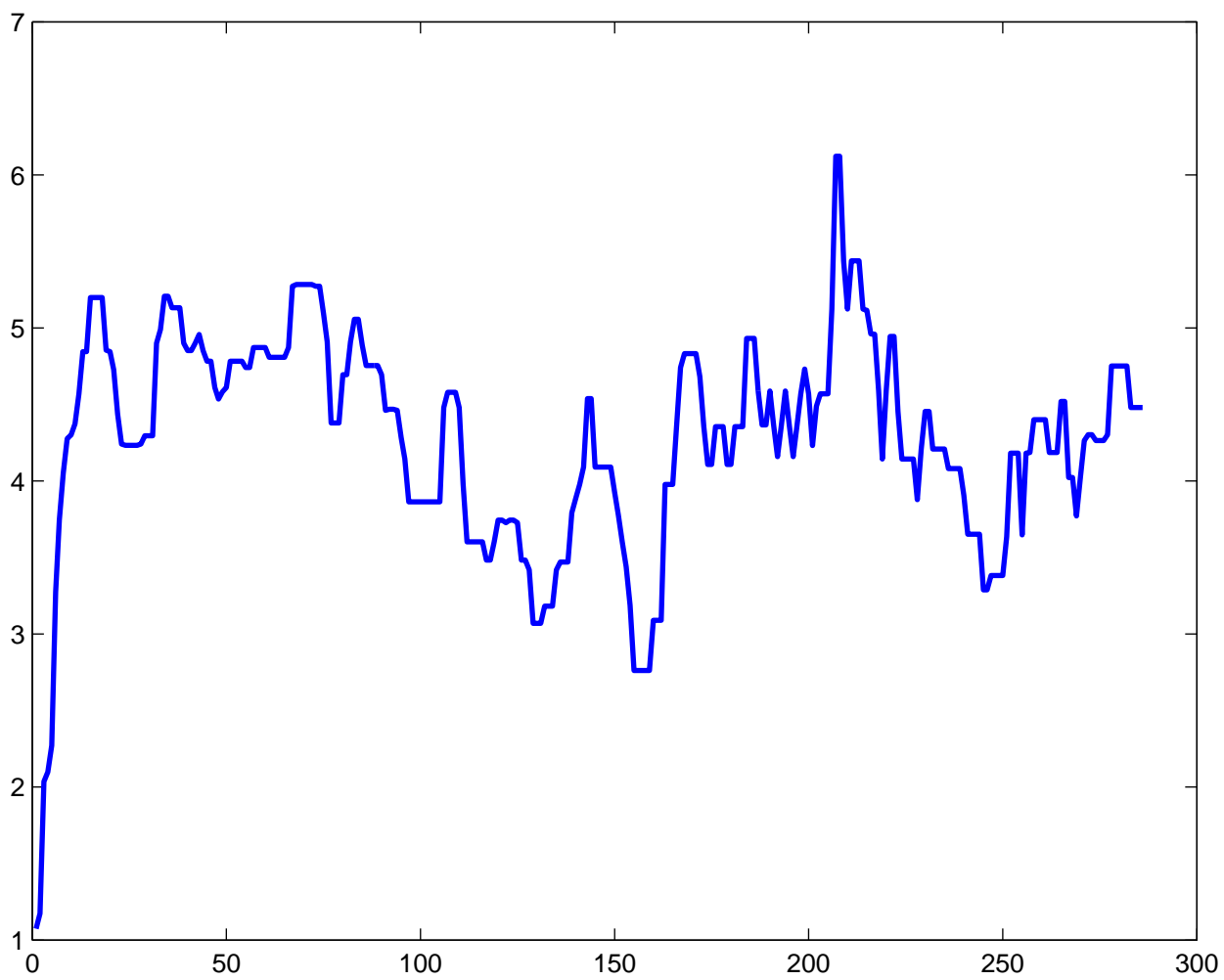

Supplement: Dataset S2 — Plots of the blood flow data of reactive hyperemia experiment for all subjects. (ZIP) [file pcbi.1003070.s002.zip › data/CTRL_C16_filt.pdf]

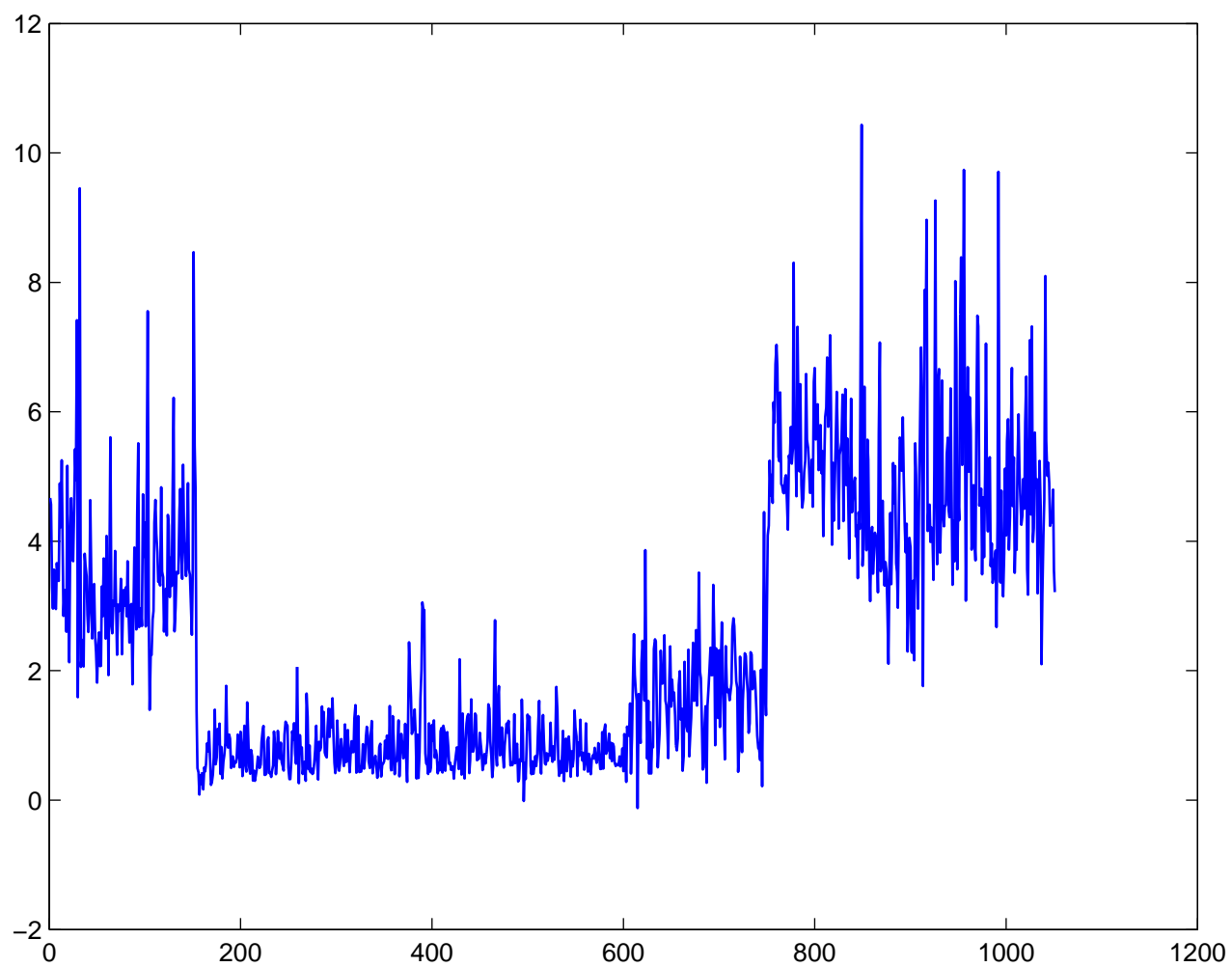

Supplement: Dataset S2 — Plots of the blood flow data of reactive hyperemia experiment for all subjects. (ZIP) [file pcbi.1003070.s002.zip › data/CTRL_C16_raw.pdf]

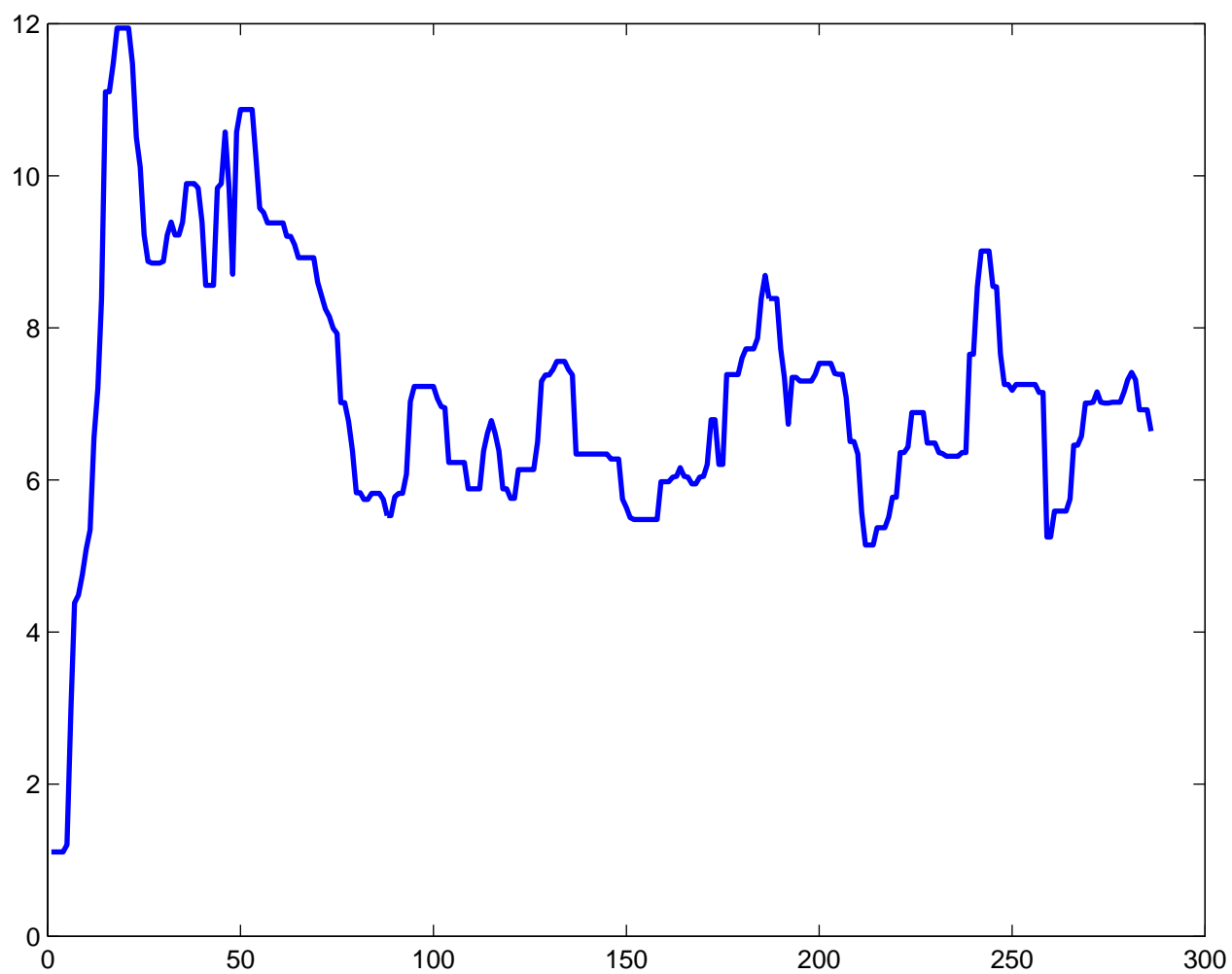

Supplement: Dataset S2 — Plots of the blood flow data of reactive hyperemia experiment for all subjects. (ZIP) [file pcbi.1003070.s002.zip › data/CTRL_C1_filt.pdf]

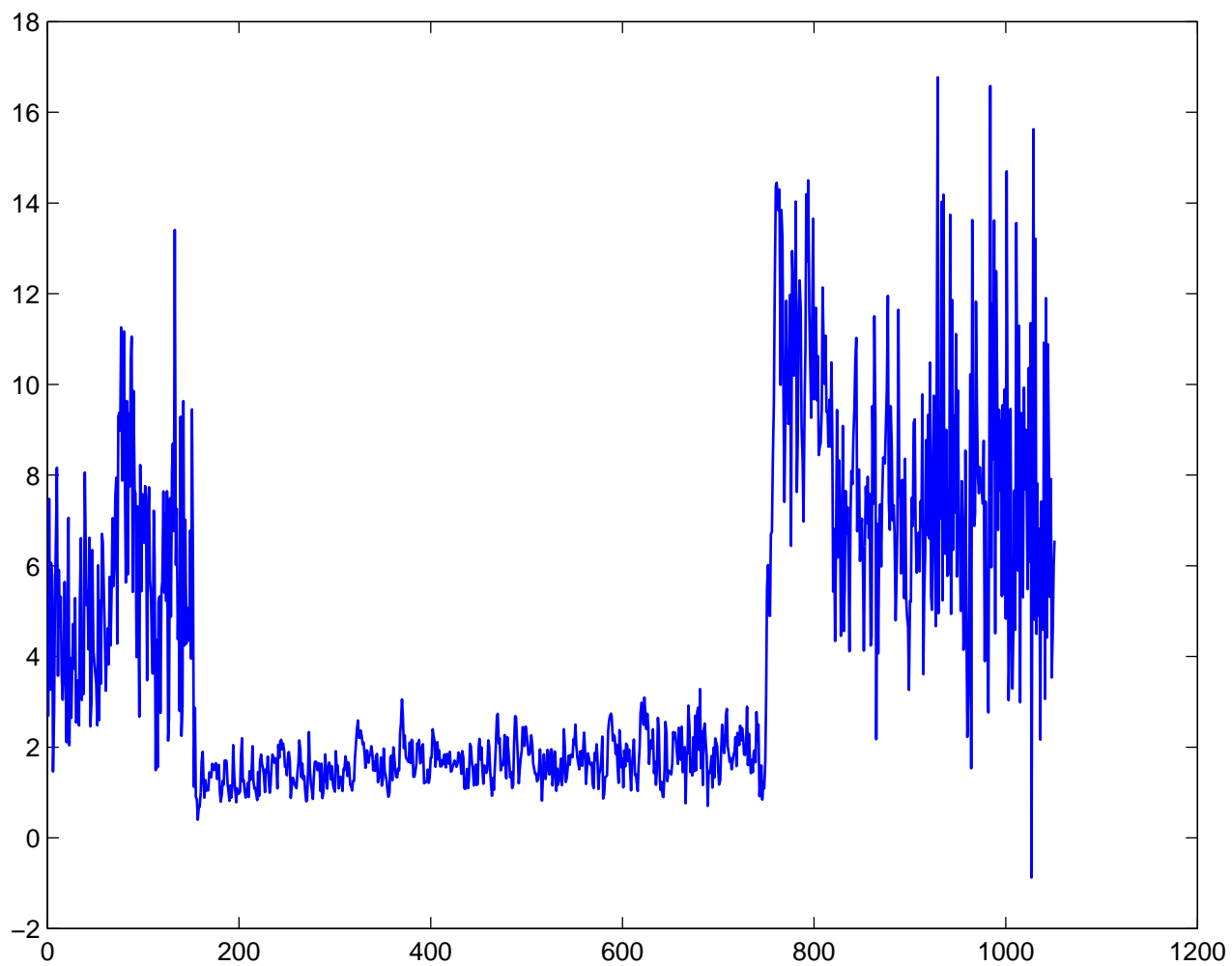

Supplement: Dataset S2 — Plots of the blood flow data of reactive hyperemia experiment for all subjects. (ZIP) [file pcbi.1003070.s002.zip › data/CTRL_C1_raw.pdf]

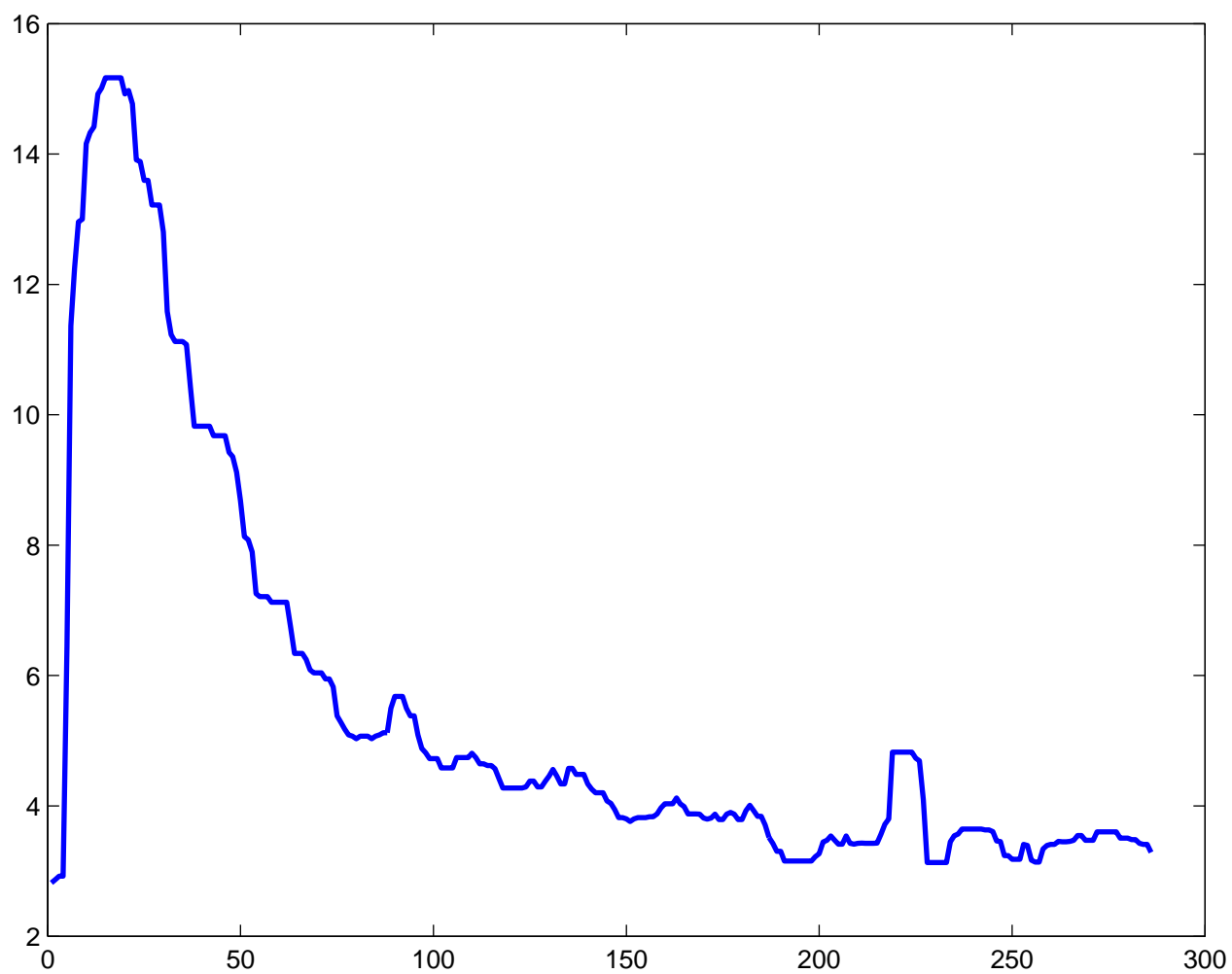

Supplement: Dataset S2 — Plots of the blood flow data of reactive hyperemia experiment for all subjects. (ZIP) [file pcbi.1003070.s002.zip › data/CTRL_C6_filt.pdf]

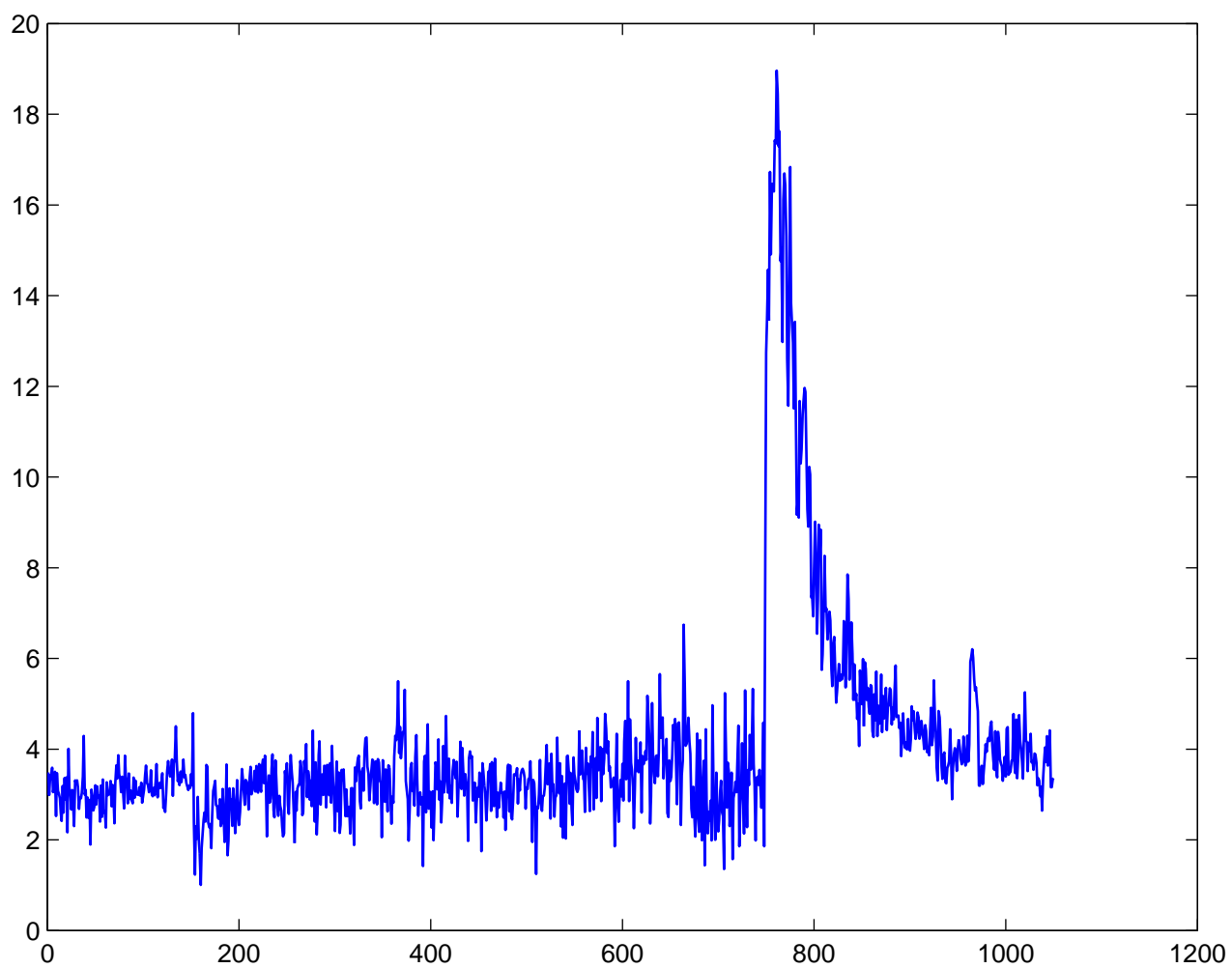

Supplement: Dataset S2 — Plots of the blood flow data of reactive hyperemia experiment for all subjects. (ZIP) [file pcbi.1003070.s002.zip › data/CTRL_C6_raw.pdf]

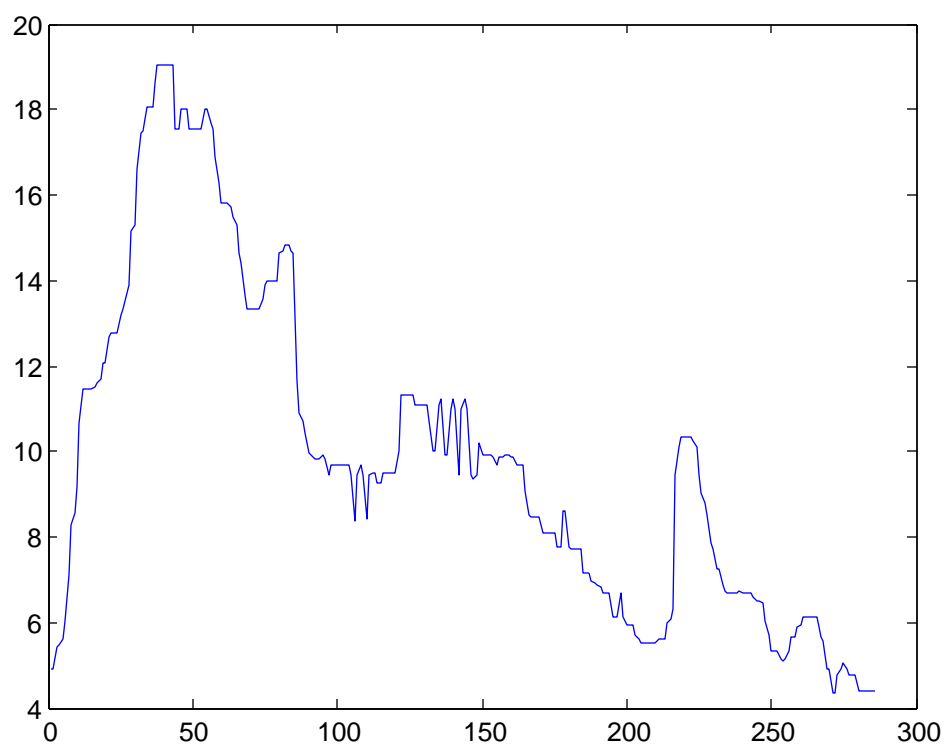

Supplement: Dataset S2 — Plots of the blood flow data of reactive hyperemia experiment for all subjects. (ZIP) [file pcbi.1003070.s002.zip › data/CTRL_C7_filt.pdf]

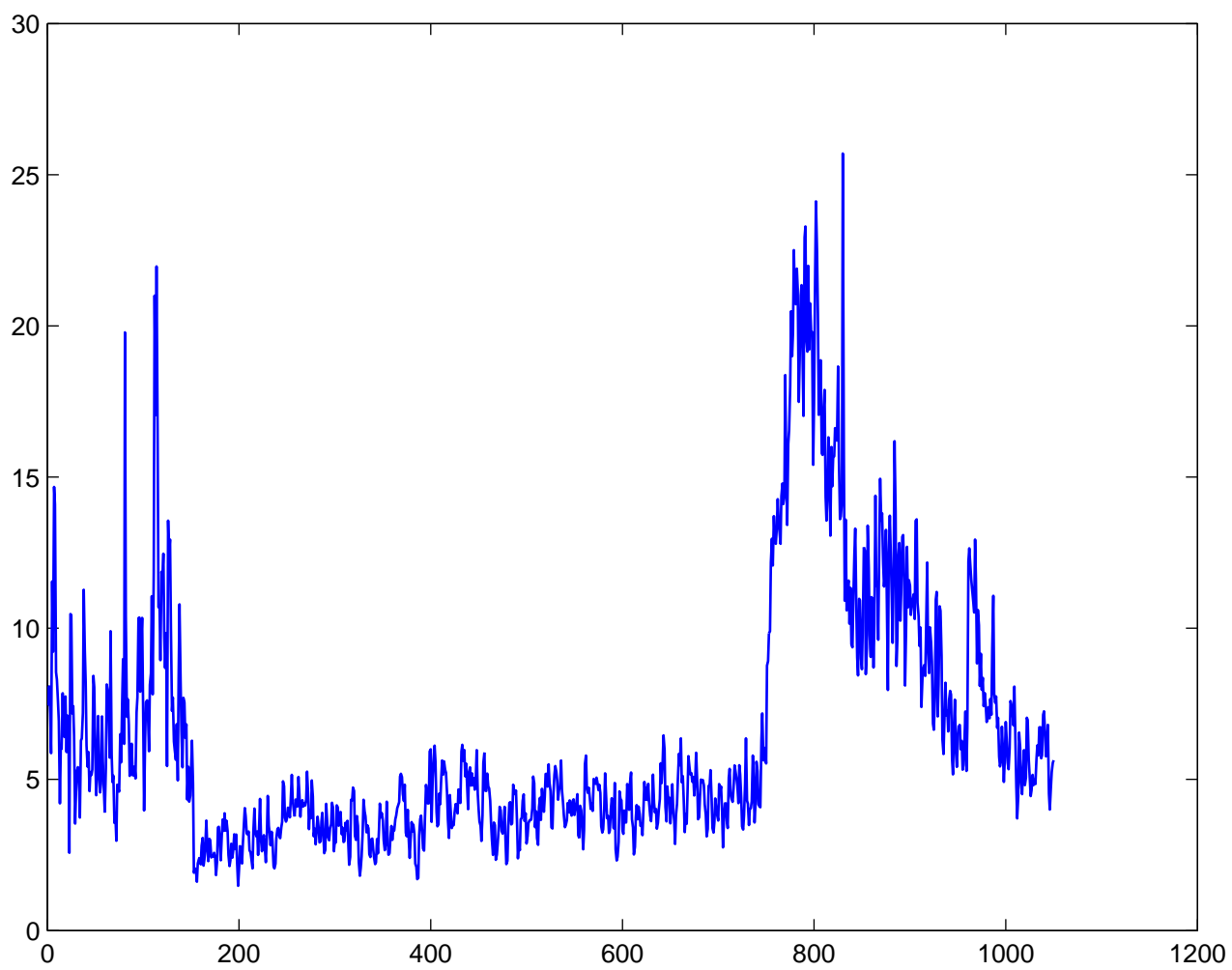

Supplement: Dataset S2 — Plots of the blood flow data of reactive hyperemia experiment for all subjects. (ZIP) [file pcbi.1003070.s002.zip › data/CTRL_C7_raw.pdf]

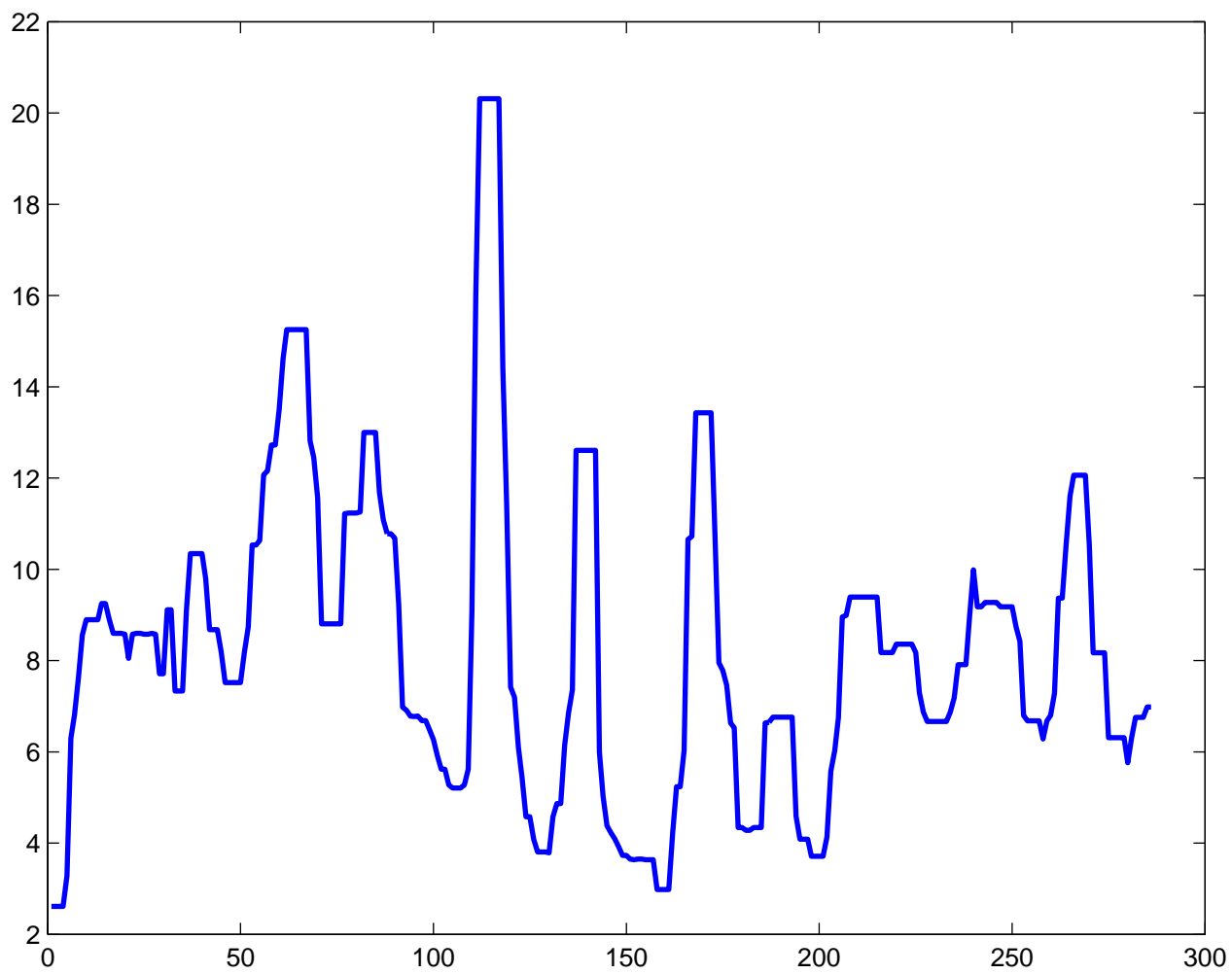

Supplement: Dataset S2 — Plots of the blood flow data of reactive hyperemia experiment for all subjects. (ZIP) [file pcbi.1003070.s002.zip › data/SCI_A10_filt.pdf]

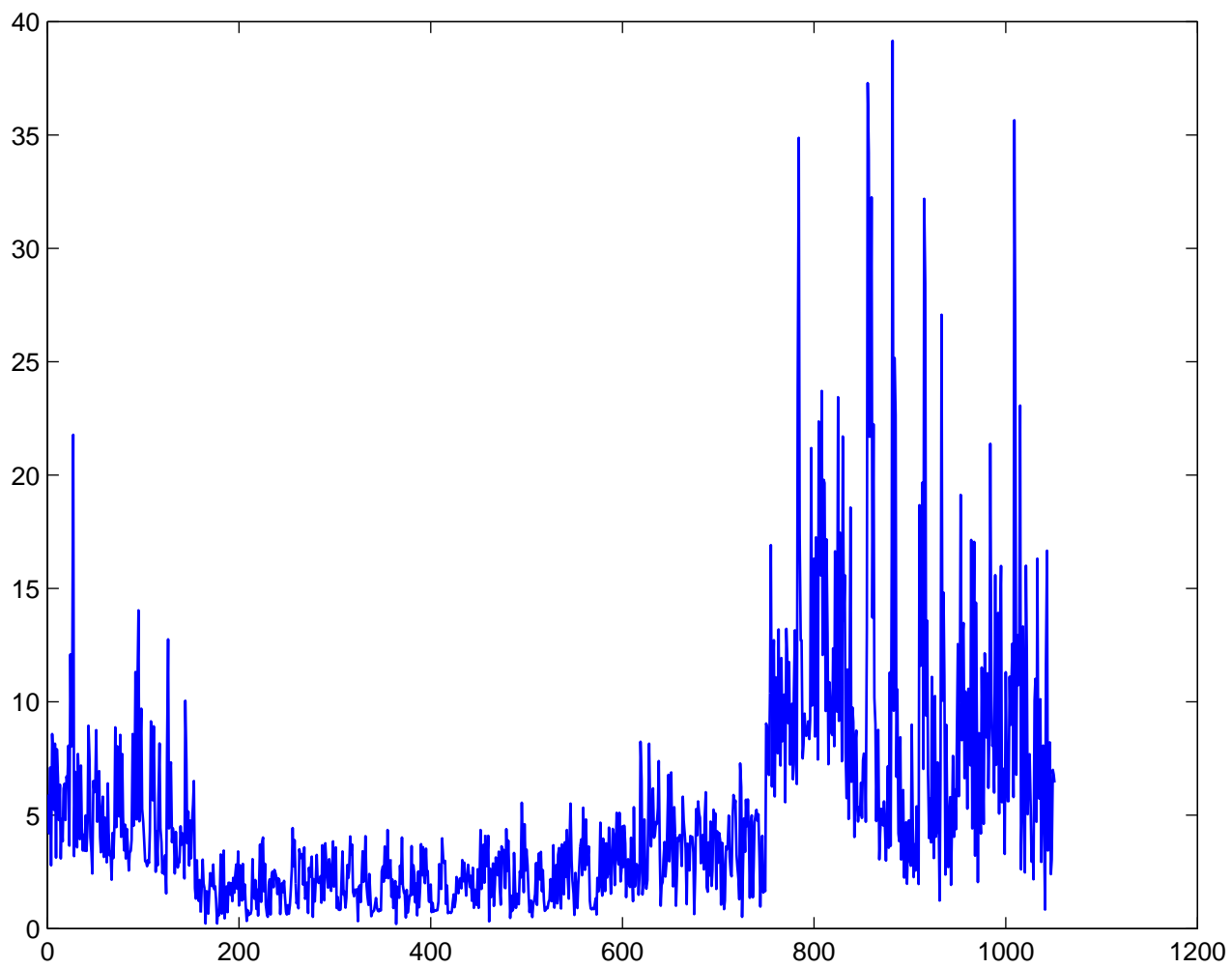

Supplement: Dataset S2 — Plots of the blood flow data of reactive hyperemia experiment for all subjects. (ZIP) [file pcbi.1003070.s002.zip › data/SCI_A10_raw.pdf]

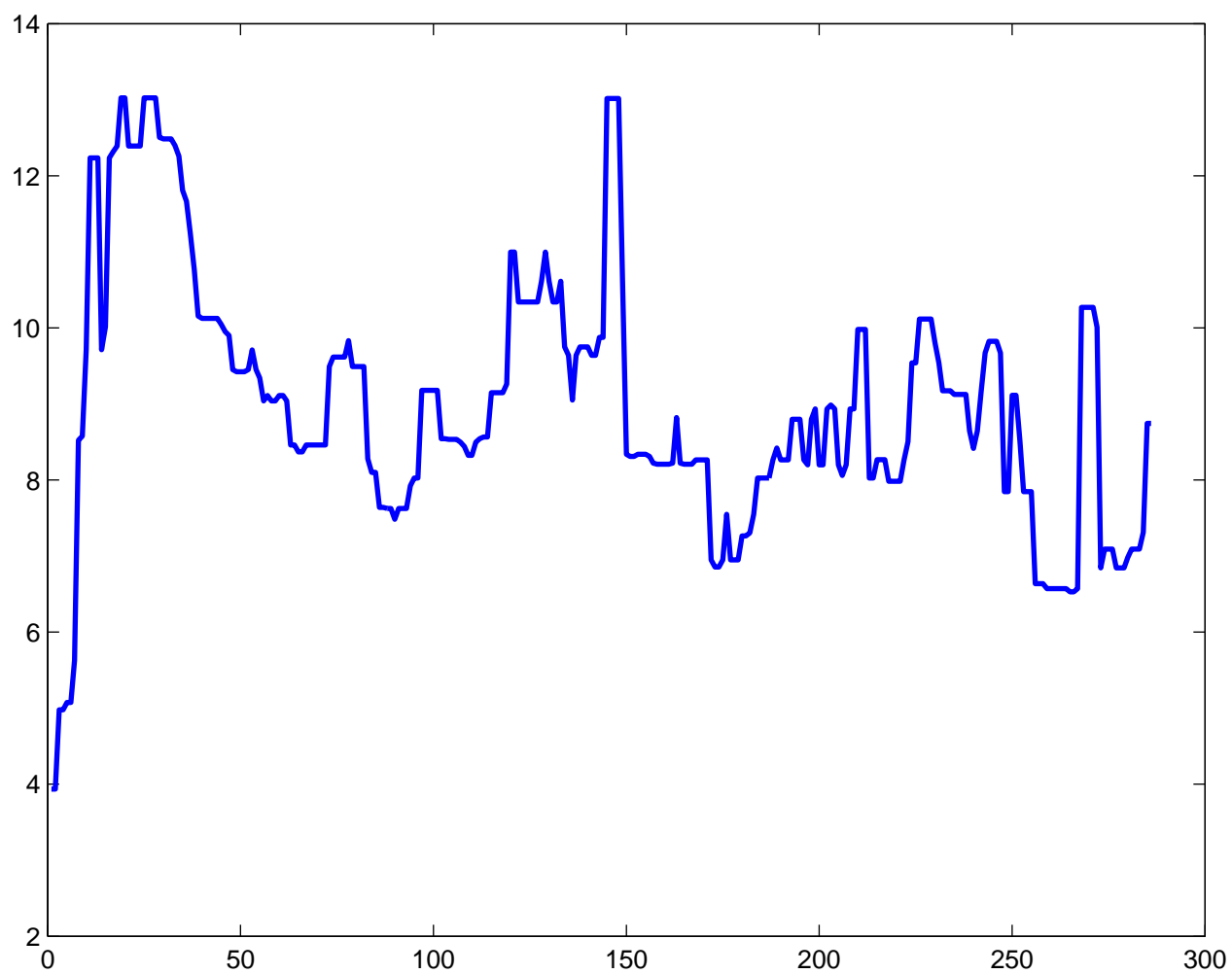

Supplement: Dataset S2 — Plots of the blood flow data of reactive hyperemia experiment for all subjects. (ZIP) [file pcbi.1003070.s002.zip › data/SCI_A14_filt.pdf]

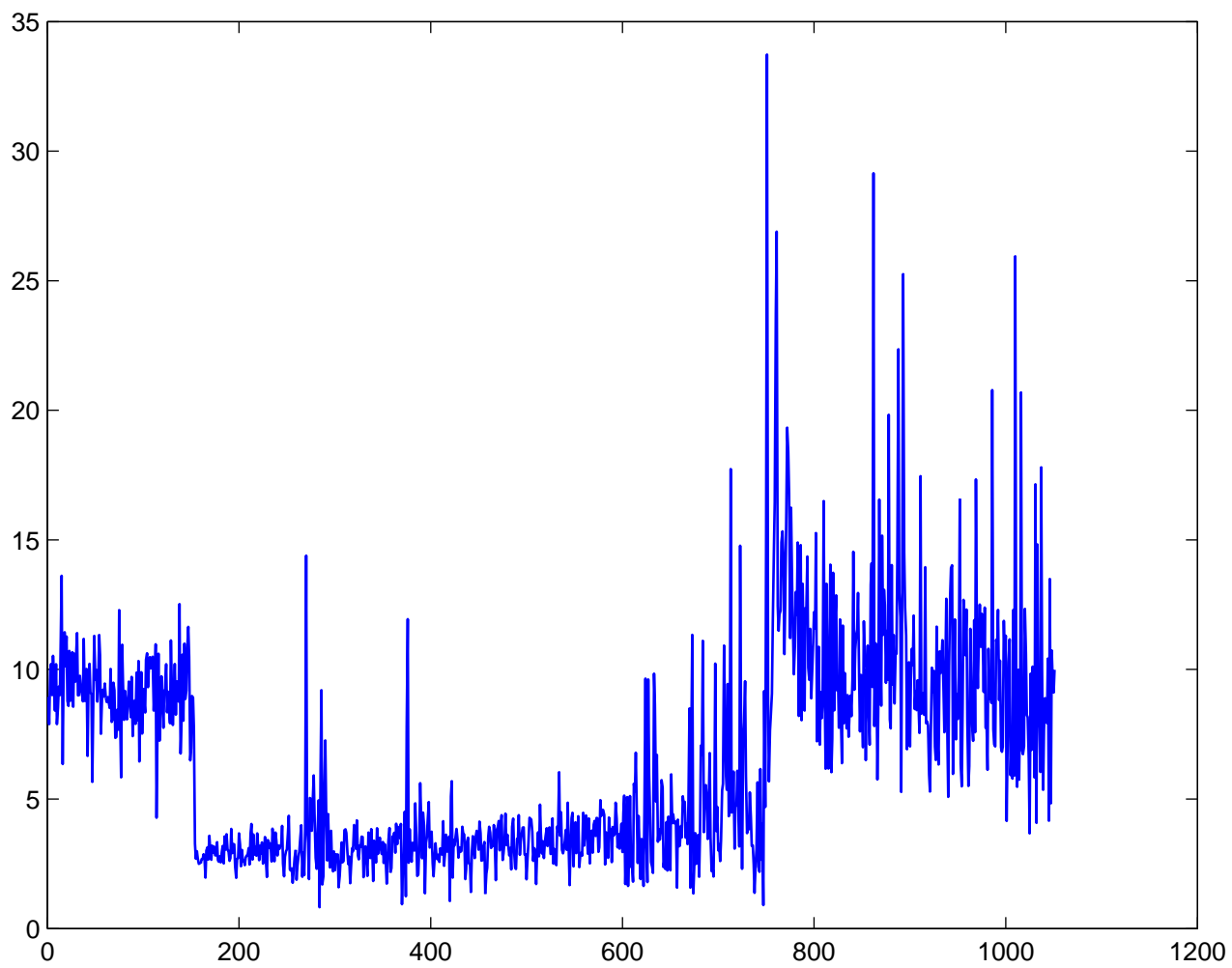

Supplement: Dataset S2 — Plots of the blood flow data of reactive hyperemia experiment for all subjects. (ZIP) [file pcbi.1003070.s002.zip › data/SCI_A14_raw.pdf]

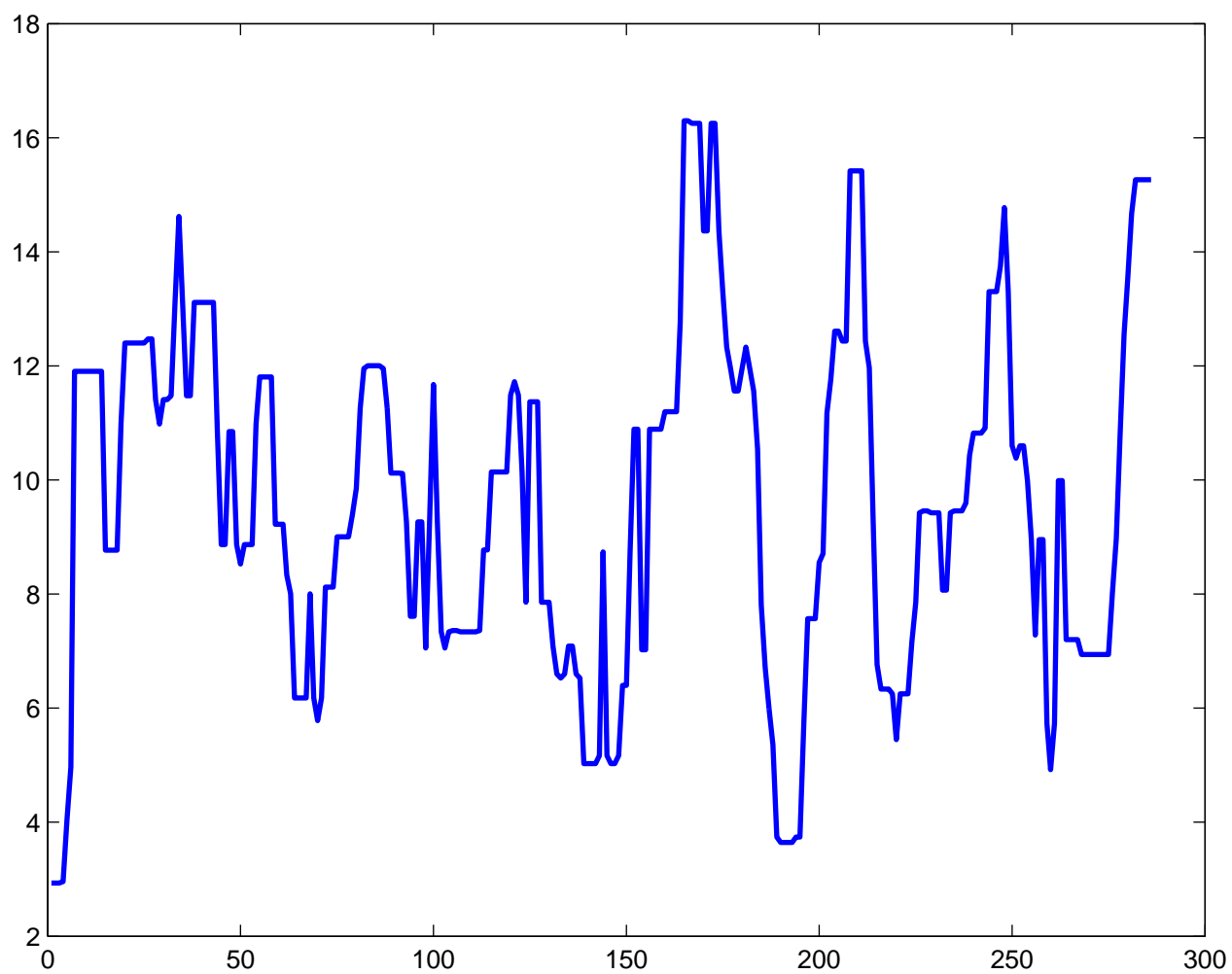

Supplement: Dataset S2 — Plots of the blood flow data of reactive hyperemia experiment for all subjects. (ZIP) [file pcbi.1003070.s002.zip › data/SCI_A15_filt.pdf]

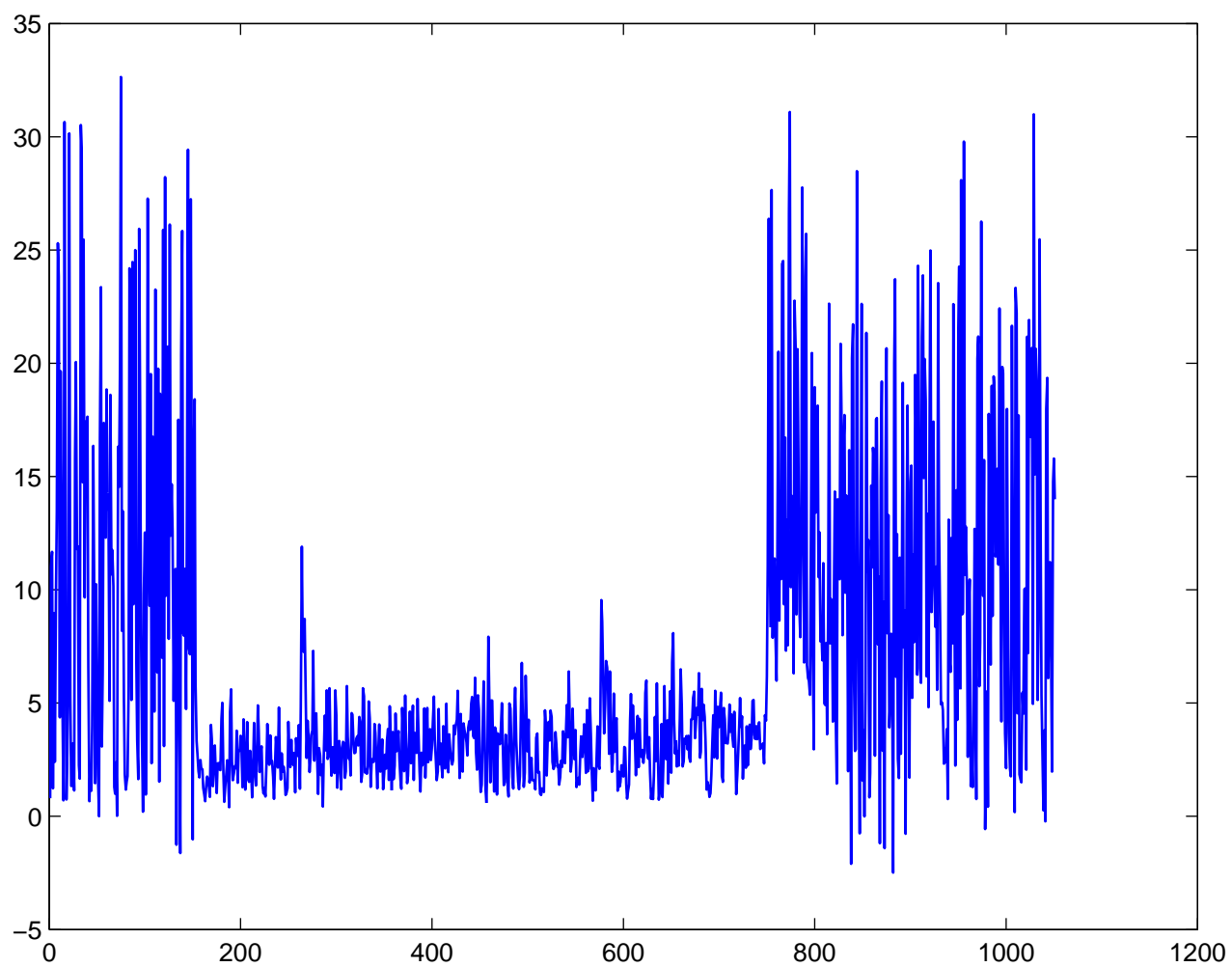

Supplement: Dataset S2 — Plots of the blood flow data of reactive hyperemia experiment for all subjects. (ZIP) [file pcbi.1003070.s002.zip › data/SCI_A15_raw.pdf]

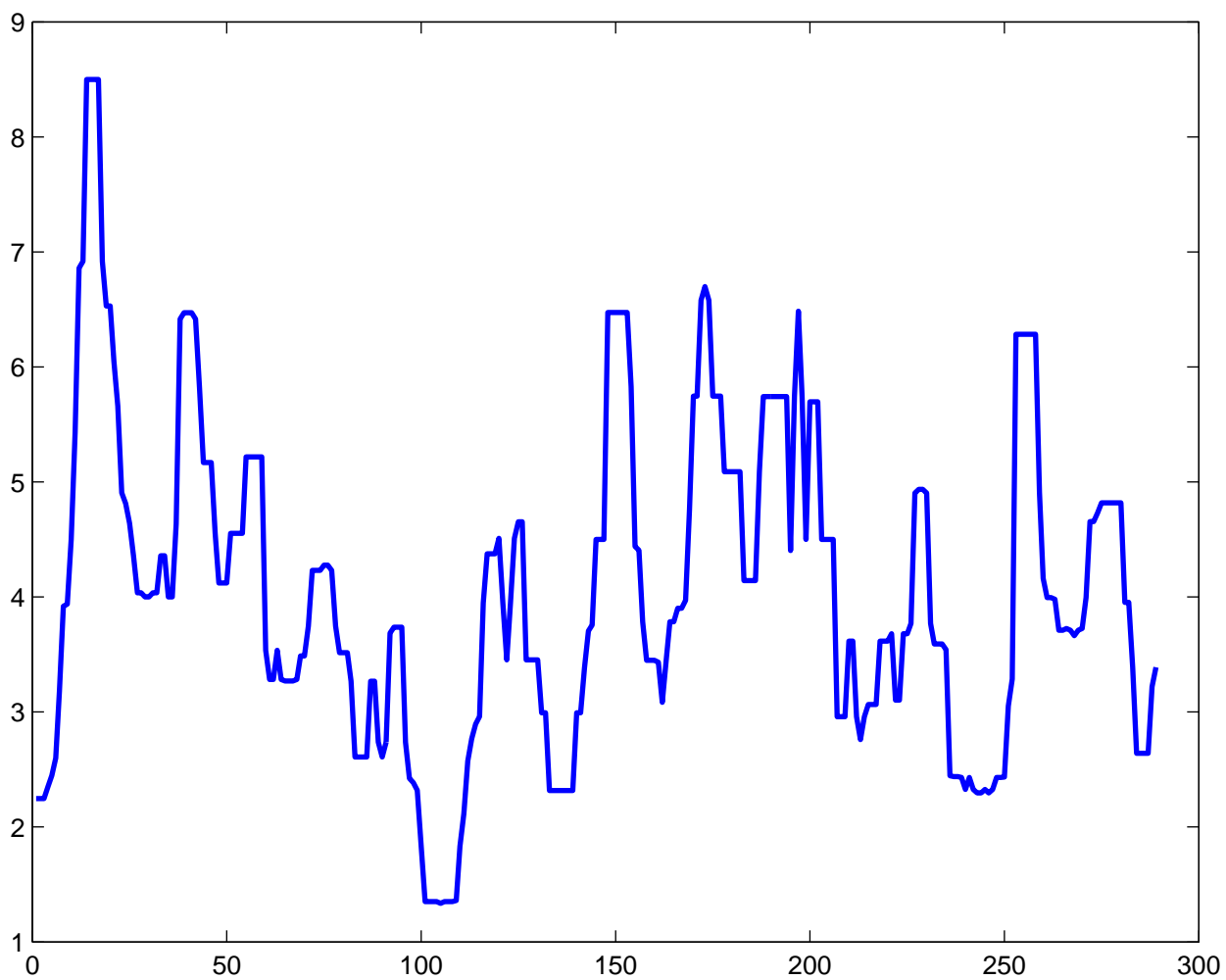

Supplement: Dataset S2 — Plots of the blood flow data of reactive hyperemia experiment for all subjects. (ZIP) [file pcbi.1003070.s002.zip › data/SCI_A7_filt.pdf]

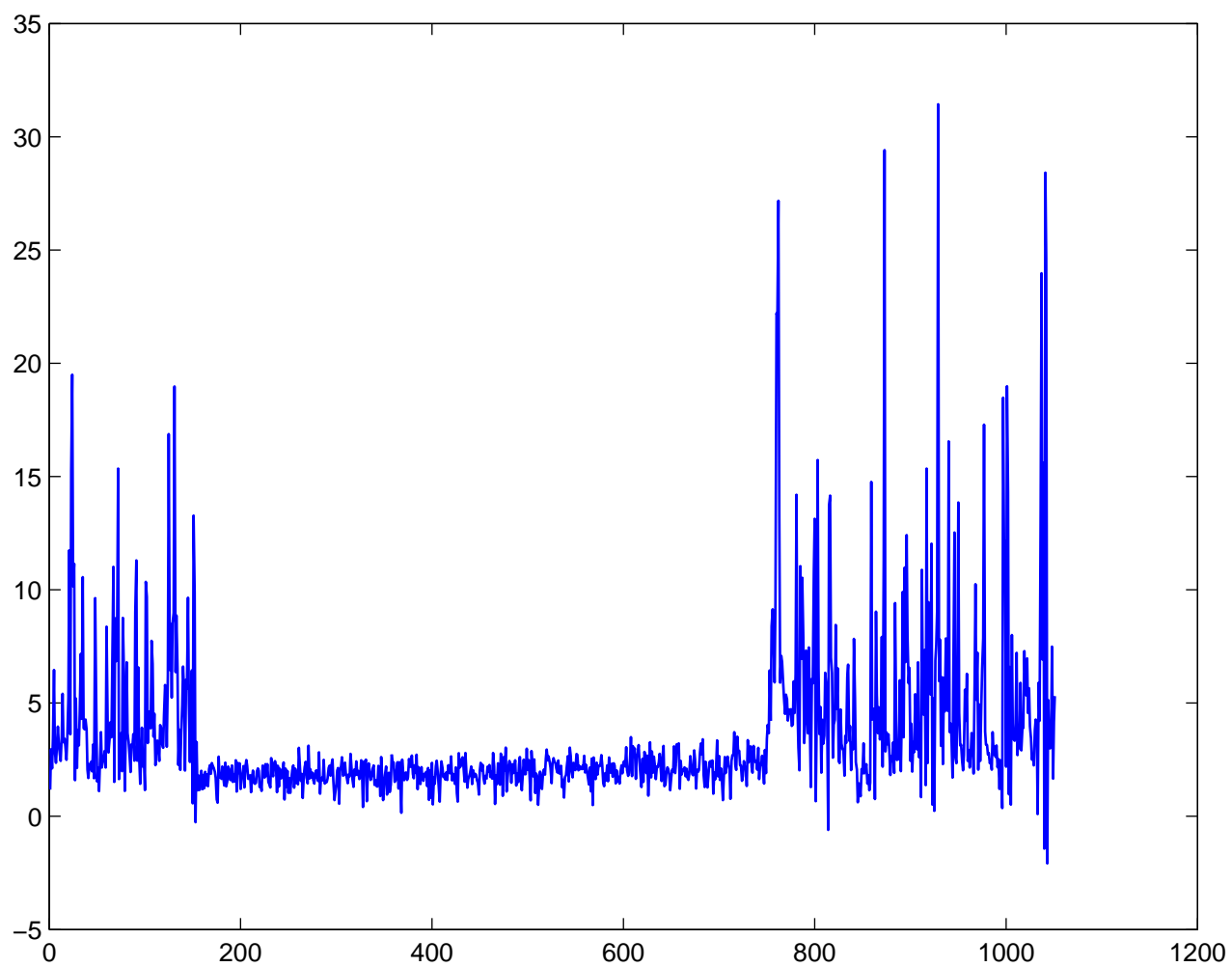

Supplement: Dataset S2 — Plots of the blood flow data of reactive hyperemia experiment for all subjects. (ZIP) [file pcbi.1003070.s002.zip › data/SCI_A7_raw.pdf]

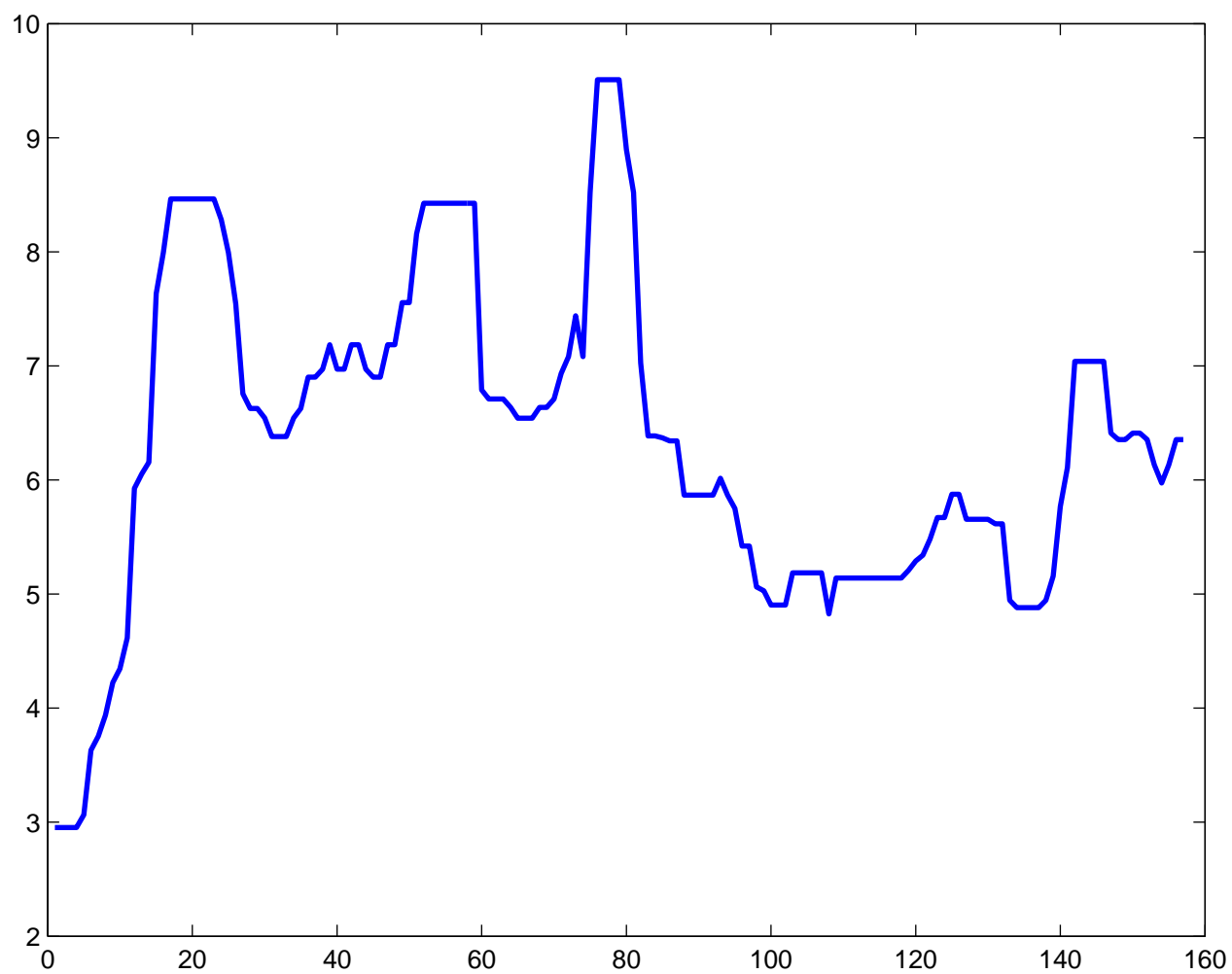

Supplement: Dataset S2 — Plots of the blood flow data of reactive hyperemia experiment for all subjects. (ZIP) [file pcbi.1003070.s002.zip › data/SCI_B3_filt.pdf]

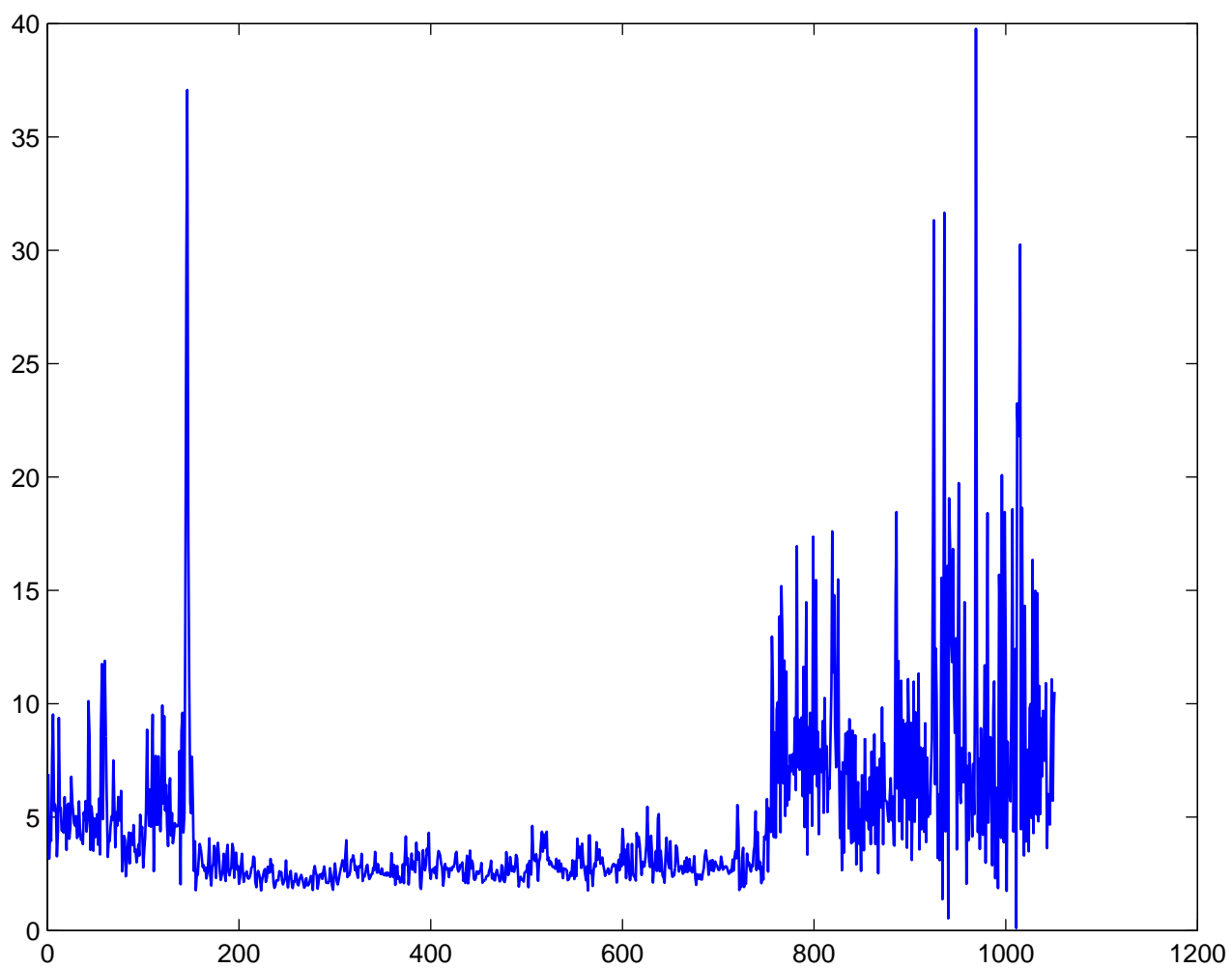

Supplement: Dataset S2 — Plots of the blood flow data of reactive hyperemia experiment for all subjects. (ZIP) [file pcbi.1003070.s002.zip › data/SCI_B3_raw.pdf]

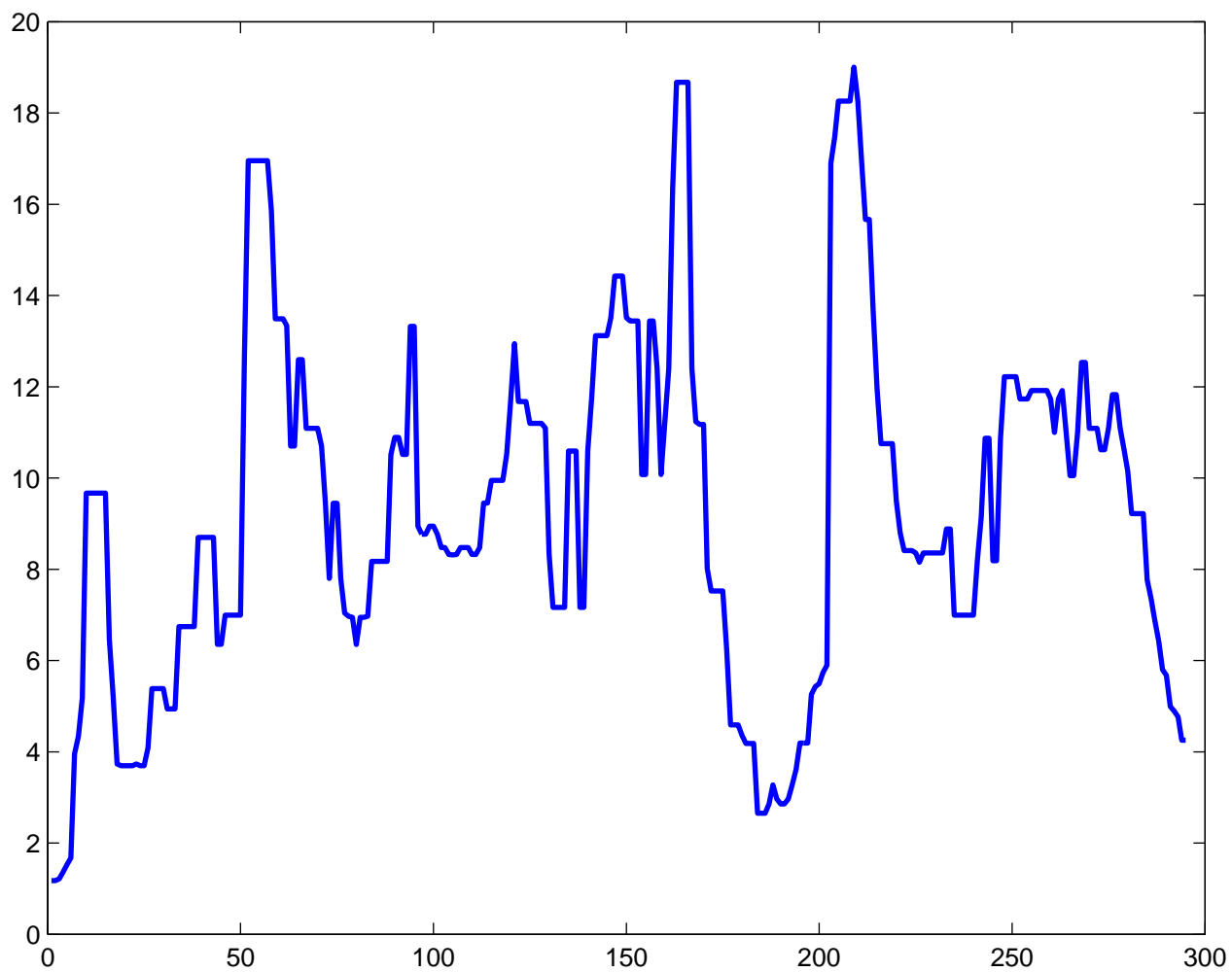

Supplement: Dataset S2 — Plots of the blood flow data of reactive hyperemia experiment for all subjects. (ZIP) [file pcbi.1003070.s002.zip › data/SCI_B5_filt.pdf]

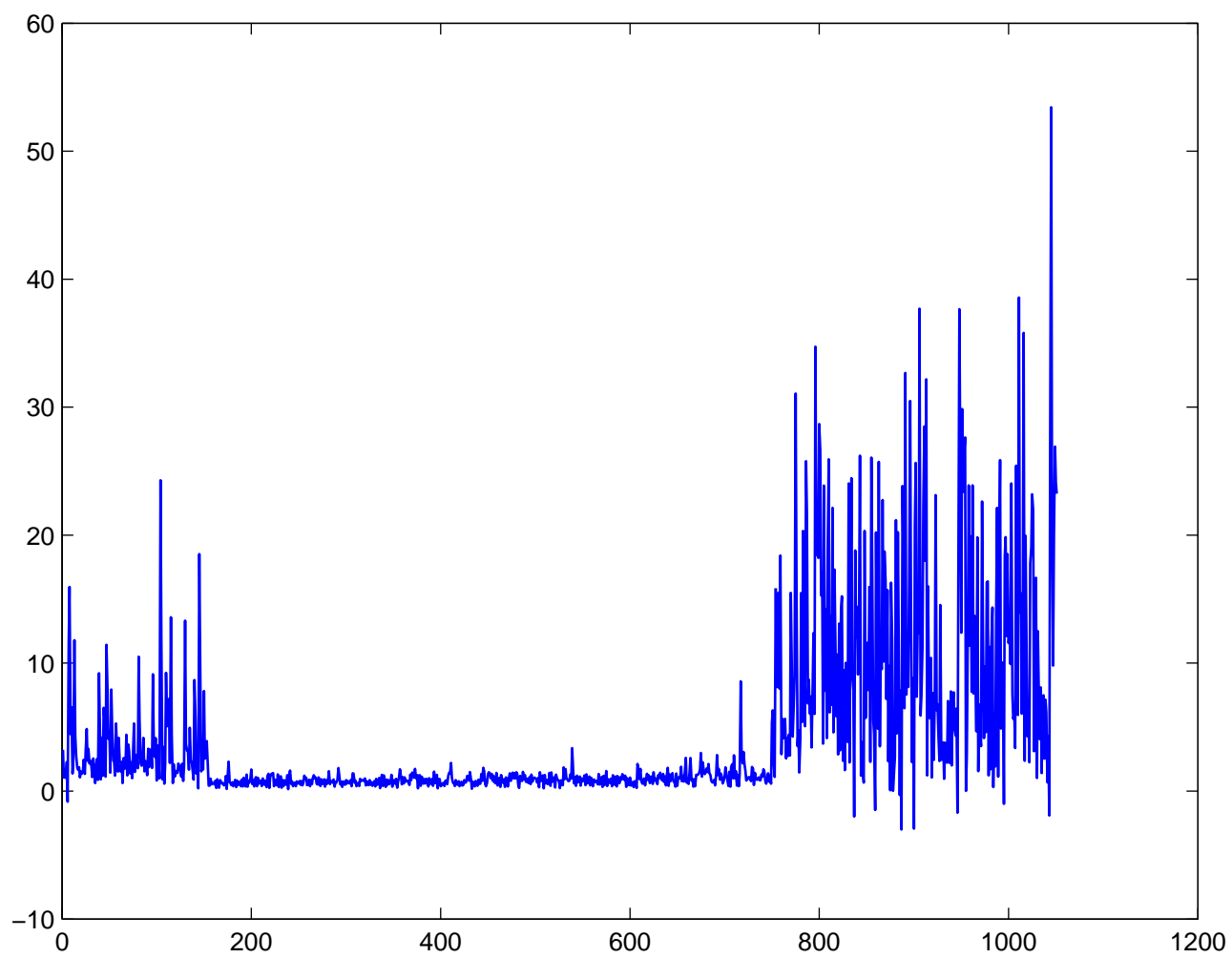

Supplement: Dataset S2 — Plots of the blood flow data of reactive hyperemia experiment for all subjects. (ZIP) [file pcbi.1003070.s002.zip › data/SCI_B5_raw.pdf]
