# Supplementary material for: Hybrid Equation/Agent-Based Model of Ischemia-Induced Hyperemia and Pressure Ulcer Formation Predicts Greater Propensity to Ulcerate in Subjects with Spinal Cord Injury
Source: PLoS Comput Biol. 2013 May 16;9(5):e1003070. doi: 10.1371/journal.pcbi.1003070 (PMC3656105; doi:10.1371/journal.pcbi.1003070)
Supplement: Text S1 — A detailed description of rules and parameters for the agent-based portion of the model. (DOCX) [file pcbi.1003070.s005.docx]

**ABM development**

The following literature on inflammation and wound healing was reviewed to identify the essential components and rules for this ABM similarly as we did in our previous work [20, 21].

1. Clark RAF (1998) Wound Repair. Overview and General Considerations. In: Clark RAF, editor. The Molecular and Cellular Biology of Wound Repair. New York: Plenum Press.

2. Cockbill S (2002) Wound: the healing process. Hospital Pharmacist 9: 255–260.

3. Martin P (1997) Wound Healing-aiming for perfect skin regeneration. Science 276: 75–81.

4. Witte MB, Barbul A (1997) General principles of wound healing. Surgical Clinics of North America 77: 509–528.

The space of model is toroidal. Several agents can occupy the same space, and macrophages use local polar coordinates for moving (heading and distance).

**ABM parameters**

| Parameter name | Default Value |
| --- | --- |
| pressure-interval | varies |
| initial-pressure | 0.5 |
| tnf-epithelial | 0.1 |
| pressure-epithelial | 0.8 |
| tgf-epithelial | 0.1 |
| tnf-production | 0.1 |
| mac-tgf-production | 0.4 |
| mac-tnf-production | 0.1 |
| macrophage-production | 0.1 |
| oxygen-production | 5.0 |
| healing-rate | 0.1 |

**ABM rules**

**Structural/functional skin cell (nominally epithelial cells)**

1. Initially created as a grid of square cells.
2. Each cell has the life value which is initialized to 100.
3. If the life is less than 0 then the cell dies.
4. Cells are damaged by pressure applied to the cell (*p*) and by TNF at the position of the cell (*tnf*). The damage is computed as follows:
   *damage = pressure-epithelial * p + tnf-epithelial * tnf.*
5. Cells are healed by TGF (*tgf*) and oxygen (*oxygen*) at the position of the cell. The amount of restored life is computed as follows:
   *(tgf-epithelial * tgf + healing-rate) * oxygen.*
6. If the life is less than 80 then the cell releases TNF (*tnf*). The amount of released TNF equals to *tnf-production.*

**Inflammatory cells (nominally macrophages)**

1. Each macrophage has a life parameter which initial value is randomly chosen in the interval [100, 150].
2. Macrophages randomly move around.
3. Macrophages are chemoattracted by TNF.
4. Macrophages release TNF. The value of released TNF at each simulation step is
   *mac-tnf-production / (1 + tgf).*
5. Macrophages release TGF. The value of released TGF at each simulation step is
   *mac-tgf-production.*
6. The life of a macrophage is decreased by 1 each time step. When the life is 0, the macrophage will die.

**Blood vessels**

1. Blood vessels are created in a sparse grid pattern.
2. If the pressure is less than 0.1 then set *V = V-max*, otherwise *V = V-min*. Compute the blood flow (*I2*) using the ODE. Compute the normal flow value (*normal-flow*) (the value of the flow when *V* is a constant and *V = V-max*).
3. The size of the blood vessel is proportional to *I2*:
   *size = I2 / (normal-flow * 2)* (if *size < 0.1* then set *size = 0.1*).
4. If there are no epithelial cells around the blood vessel, then the blood vessel is destroyed.
5. Create a macrophage with probability:
   *macrophage-production * size * 100%.*
6. Release oxygen:
   *oxygen-production * size.*

**General rules**

1. TNF is diffused with the diffusion parameter 0.9.
2. TGF is diffused with the diffusion parameter 0.9.
3. Oxygen is diffused with the diffusion parameter 1.
4. TNF is evaporated with the parameter 0.9.
5. TGF is evaporated with the parameter 0.9.
6. Oxygen is evaporated with the parameter 0.95.
7. Apply *initial-pressure* amount of pressure for the *pressure-interval* ticks and then release the pressure for the same amount of time. Repeat indefinitely.
